# An extended dsRBD is required for post-transcriptional modification in human tRNAs

**DOI:** 10.1093/nar/gkv989

**Published:** 2015-10-01

**Authors:** Charles Bou-Nader, Ludovic Pecqueur, Damien Bregeon, Amina Kamah, Vincent Guérineau, Béatrice Golinelli-Pimpaneau, Beatriz G. Guimarães, Marc Fontecave, Djemel Hamdane

**Affiliations:** 1 Laboratoire de Chimie des Processus Biologiques, CNRS-UMR 8229, Collège De France, France, 11 place Marcelin Berthelot, 75231 Paris Cedex 05, France; 2 Sorbonne Universités, UPMC Univ. Paris 06, IBPS, UMR8256, Biology of Aging and Adaptation, F-75005 Paris, France; 3 Université de Lille-Nord de France, CNRS UMR 8576, Institut Fédératif de Recherches 147, Villeneuve d'Ascq, France; 4 Institut de Chimie des Substances Naturelles, Centre de Recherche de Gif, CNRS, 1 avenue de la Terrasse, 91198 Gif-sur-Yvette, France; 5 Synchrotron SOLEIL, L'Orme des Merisiers, Saint-Aubin, 91190 Gif-sur-Yvette, France

## Abstract

In tRNA, dihydrouridine is a conserved modified base generated by the post-transcriptional reduction of uridine. Formation of dihydrouridine 20, located in the D-loop, is catalyzed by dihydrouridine synthase 2 (Dus2). Human Dus2 (HsDus2) expression is upregulated in lung cancers, offering a growth advantage throughout its ability to interact with components of the translation apparatus and inhibit apoptosis. Here, we report the crystal structure of the individual domains of HsDus2 and their functional characterization. HsDus2 is organized into three major modules. The N-terminal catalytic domain contains the flavin cofactor involved in the reduction of uridine. The second module is the conserved α-helical domain known as the tRNA binding domain in HsDus2 homologues. It is connected via a flexible linker to an unusual extended version of a dsRNA binding domain (dsRBD). Enzymatic assays and yeast complementation showed that the catalytic domain binds selectively NADPH but cannot reduce uridine in the absence of the dsRBD. While in Dus enzymes from bacteria, plants and fungi, tRNA binding is essentially achieved by the α-helical domain, we showed that in HsDus2 this function is carried out by the dsRBD. This is the first reported case of a tRNA-modifying enzyme carrying a dsRBD used to bind tRNAs.

## INTRODUCTION

tRNA maturation requires extensive processing and a large number of chemical modifications (1). 5,6-dihydrouridine (D) is one of the most abundant modified bases in tRNAs. In tRNA, this non-aromatic base is found at unique or multiple site(s) predominantly in the so-called D-Loop (2). D results from the reduction of the 5,6-uridine double bond, which leads to a non-planar base moiety and thus the nucleoside does not participate in base stacking (3–6). It instead promotes tertiary interactions at the elbow region of tRNAs. A role of D in conformational flexibility is consistent with its high level in tRNAs from psychrophilic bacteria, in which it provides an obvious benefit under conditions where thermal motion, enzymatic reaction kinetics and intermolecular interactions are compromised (7). Recent studies have contributed to uncover the physiological role of D, notably by showing that this modification may act as a tRNA quality control marker. Indeed, deficiency of D, as well as of other modified bases, results in enhanced tRNA degradation, at rates approaching those seen for mRNA degradation (8).

The genes encoding dihydrouridine synthases (Dus) have been identified in yeast *Saccharomyces cerevisiae* and *Escherichia coli* (9–12). These enzymes are homologous to dihydroorotate dehydrogenases and dihydropyrimidine dehydrogenases and they use a flavin mononucleotide (FMN) to catalyze hydride transfer from NAD(P)H to the uridine substrate (9). A recent biochemical study established an enzymatic mechanism for *S. cerevisiae* Dus2 (Dus2p), which is responsible for formation of the widely conserved D20 (13). Dus2p can bind the tRNA substrate and catalyze uridine reduction efficiently only if tRNA contains prior modifications. Three-dimensional structures have been obtained only in the case of bacterial Dus proteins (14–16). They share a similar scaffold composed of two subdomains, an N-terminal α/β-barrel carrying the catalytic site and a C-terminal α-helical domain implicated in tRNA binding. Interestingly, *Thermus thermophilus* Dus (*Tth*Dus) has recently been crystallized in complex with tRNA (16). In this structure, the enzyme interacts extensively with the D-arm and recognizes the elbow region formed by the interaction between the T- and D-loops in tRNA. Consistently, it has been proposed that Dus catalyzes hydride transfer after the tertiary structure of the tRNA has been controlled, especially the elbow region, which could be seen as a ‘hallmark’ of the completion of the L shape formation (16). Recently, a crystal structure of E. *coli* DusC in complex with tRNA, targeting U16, has revealed a completely different tRNA binding mode (17).

Increase of D content has long been observed in tRNAs from malignant human tissues (18). This has been intriguing until a recent study showed a clear correlation between high expression levels of a human protein named HsDus2, homologous to Dus2p, and the potentiality to develop a non-small cell lung cancer (NSCLC) (19). Moreover, transfection of NSCLC cells with a specific siRNA against HsDus2 resulted in decreased HsDus2 levels and cell growth inhibition.

A direct interaction between HsDus2 and glutamyl-prolyl tRNA synthetase might activate translation processes and contribute to cell growth (19). HsDus2 has also been shown to interact with a double-stranded RNA-activated protein kinase (PKR) (20), an interferon-induced protein involved in regulation of antiviral innate immunity, stress signaling, cell proliferation and programmed cell death (21). PKR interacts with HsDus2 via its own double-stranded RNA binding domain (dsRBD) and the formation of the complex results in the inhibition of the kinase activity, thereby escaping apoptosis. An efficient activator of PKR, PACT, has also been shown to interact with HsDus2, offering an additional effective PKR inhibition pathway (20). It is thus clear that, upon up-regulation, HsDus2 plays a role in cell proliferation and apoptosis.

For a more substantial understanding of the molecular function of HsDus2, its chemical mechanism, the interaction with its various macromolecular partners and for the rational design of specific inhibitors, a three-dimensional structure would be highly valuable. However, there is no such structure available so far as only the crystallization and preliminary X-ray characterization of the HsDus2 catalytic domain have been reported (22). HsDus2 and bacterial Dus share less than 25% sequence identity. In addition, several inserted and deleted regions are present along the HsDus2 sequence (see alignments in Supplementary Figure SI), suggesting that the human enzyme structure should have unique features.

**Figure 1.**
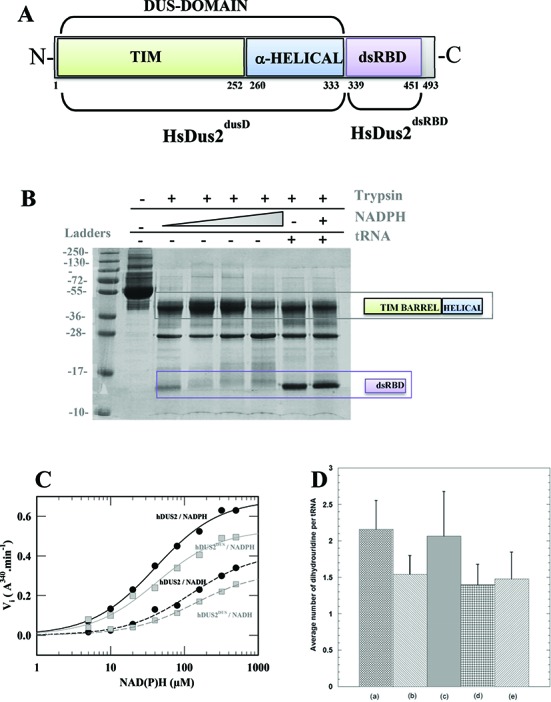
Structural and functional modularity of HsDus2. (**A**) Domain architecture of HsDus2. The boundaries of each domain are based from our mild proteolysis coupled to MS-MALDI analysis as well as crystal structures. The numbering of the residues does not take into account the first histidine of the His-Tag but it starts at the N-terminus methionine. While the TIM barrel (green) and the α-helical sub-domain (blue) are strictly conserved among the Dus family, the dsRBD is found only in Dus2 from animals. The combination of the TIM barrel and the α-helical sub-domain forms the so-called ‘Dus domain’ referred as HsDus2^dusD^ in the text. The C-terminal extension ranging from 451–493 is predicted as an unstructured region. (**B**) Mild trypsinolysis pattern of HsDus2 with and without its substrates. (**C**) Oxidation of NADH and NADPH by HsDus2 and HsDus2^dusD^ under steady state conditions. (**D**) *In vitro* quantification of average dihydrouridine content per tRNA in (a) wild type yeast bulk tRNA, (b) bulk tRNA extracted from yeast deleted of *dus2* (bulk Δ*dus2* tRNA), (c) bulk Δ*dus2* tRNA in the presence of HsDus2 and NADPH, (d) bulk Δ*dus2* tRNA in the presence of HsDus2^dusD^ and NADPH and (e) bulk Δ*dus2* tRNA in the presence of HsDus2^dsRBD^ and NADPH. The resulting errors are obtained from the average of 5 independent experiments.

In the following, the combination of these two sub-domains (i.e TIM barrel and the helical domains) is referred as the Dus domain. Bioinformatics studies predicted an additional domain for HsDus2 (Figure 1A) (20,23). The human enzyme conserves the ‘bona fide’ Dus domain that spans the first 330 amino acids, while a presumably unstructured linker (residues 331–367) connects the latter to an unprecedented double-stranded RNA binding domain (dsRBD) formed by residues 368 to 433. The C-terminal region (434–489) is predicted as a disordered extension.

In this work, we report for the first time the crystal structure of all the domains of HsDus2 and their functional characterization.

## MATERIALS AND METHODS

### Preparation of the proteins and RNAs

All the recombinant proteins were cloned, expressed and purified according the detailed procedure presented in SI Text. After Ni^2+^-NTA agarose affinity column (Qiagen), the proteins were further purified on a Superdex S200 10/300 GL or S200 16/600 column (GE Healthcare Inc.) using 25 mM Tris pH 8, 150 mM NaCl buffer. The proteins were analyzed by SDS-PAGE gel and stored at −80°C. The preparation of bulk and *in vitro* transcripts tRNAs are described in the SI Text. All the rationale and the procedure used to identify and delineate HsDus2 domains are described in the SI Text.

### Yeast complementation and MS analysis of tRNAs containing D20

Sequences corresponding to HsDus2, HsDus2^dusD^ and HsDus2^dsRBD^ were cloned between BamHI and NotI in pCM190. The yeast Kozak sequence (AAAAAA) was also inserted 5′ of each ATG. Bulk tRNA was extracted from yeast strains BY4741 (MATa; *his3*Δ 1; *leu2*Δ 0; *met15*Δ 0; *ura3*Δ 0) and its derivative Δ*dus2* (MATa; *his3*Δ 1; *leu2*Δ 0; *met15*Δ 0; *ura3*Δ 0; YNR015w::kanMX4). Cells were grown in 500 ml of YPD (peptone 2%; yeast extract 1% and glucose 2%) to an OD600 of 0.8. Pelleted cells were washed twice in 20 ml of TMN (10 mM Tris–HCl (pH 7.5), 10 mM MgCl_2_, 0.15 M NaCl). The final pellet was resuspended in 20 ml of TMN, 20 ml of acidic phenol (pH 4.5) and incubated for 20 min at room temperature on a rotating wheel. After centrifugation, the aqueous phase was recovered, supplemented with LiCl to a final concentration of 0.8 M and incubated overnight at 4°C to precipitate high molecular mass molecules. The precipitate was eliminated by centrifugation and the supernatant was supplemented with two volumes of 100% ethanol and incubated at −20°C for 2 h to precipitate tRNAs. After centrifugation, pelleted tRNAs were washed twice in 70% ethanol and resuspended in 1 ml of RNAse-free water. tRNAs were desalted and concentrated four times to 50 μl in Centricon YM-3 devices (Millipore) using 100 mM ammonium acetate (pH 5.3) as a final buffer. For mass spectrometry analysis, about 50 μg of tRNAs were digested with 10 μg of RNAse A (Euromedex), which cleaves after C and U and generates 3′-phosphate nucleosides, in a final volume of 10 μl at 37°C for 4 h. One microliter of digest was mixed with 9 μl HPA (40 mg/ml in water: acetonitrile 50:50) and 1 μl of the mixture was spotted on the MALDI plate and air-dried (‘dried droplet’ method) as described previously (24). MALDI-TOF MS analyses were performed directly on the digestion products using an UltrafleXtreme spectrometer (Bruker Daltonique, France). Acquisitions were performed in positive ion mode.

### Activity assays

The ability of HsDus2 and its subdomains to oxidize NADH and/or NADPH under steady state conditions was determined in presence of menadione, as final electron acceptor, in 50 mM K_2_HPO_4_ pH 7.5, 150 mM NaCl and 20% glycerol. Assays were performed using 1 μM protein, 200 μM menadione and various concentrations of NAD(P)H ranging from 5 to 250 μM. The amount of NAD(P)H oxidized was monitored by following the decrease of absorbance at 343 nm (ϵ_343_ = 6.21 mM^−1^ cm^−1^). The initial rate versus NAD(P)H concentration was analyzed according to Michaelis–Menten formalism. Dihydrouridine formation was quantified according to a previously established colorimetric method based on the quantification of acyclic ureido group formed by alkaline cleavage of the dihydrouridine ring (25). See the SI Text for more details.

### Gel shift assay

Protein–tRNA complexes were reconstituted *in vitro* by mixing 10 μM tRNA with increasing amounts of protein (from 1 to 136 μM) in buffer D to a final volume of 20 μl. The samples were incubated for 20 min at 25°C and then 1% of bromophenol blue was added to the mixture. The samples were loaded on native gel acrylamide/Bis (37.5:1) and the electrophoresis was carried out for 2 h at 4°C and 100 volts. RNAs were colored with 0.1% toluidine solution. Intensities of the RNA bands were quantified on a Gel Doc™EZ Image (Bio RAD) with ImageLab 5.0 software.

### Crystallization, data collection and structure determination

Crystals of both HsDus2 domains were obtained at 292 K by hanging drop vapor diffusion method (see SI Text). Data collection and processing statistics are given in Supplementary Table S1.

## RESULTS AND DISCUSSION

### Human dihydrouridine synthase 2 is a modular enzyme

To examine and confirm the HsDus2 modularity experimentally, the recombinant enzyme (see purification and characterization of HsDus2 in SI Text) was subjected to mild trypsinolysis. In the presence of low concentrations of trypsin, the full-length enzyme (∼ 55 kDa) was digested into two main fragments, as shown by two major distinct bands at 28 and 38 kDa as well as an additional barely visible band at ∼14 kDa, in electrophoresis gels (Figure 1B). We tentatively assigned the 38 kDa band to the catalytic Dus domain whereas the 14 kDa to the C-terminus dsRBD. To confirm this assignment and to delineate the correct boundaries of each domain, both bands were excised and digested entirely with trypsin or endoproteinase AspN in the gel according a standardized protocol, and the resulting fragments subjected to MALDI peptide mass finger printing analysis (Supplementary Figure S2). The major band presents protein coverage of 53% with peptides spanning from Leu14–Arg333 of the Dus catalytic domain. Analysis of the lower molecular weight band gave protein coverage of 33% with the presence of peptides from the catalytic domain that are likely due to a contamination but it clearly showed the peptides region from Thr339 to Lys451, residues of the dsRBD. Interestingly, these latter boundaries are somewhat different from those predicted by bioinformatics tools notably with an extension of 8 and 18 amino acids at the N- and C-terminus, respectively, indicating that the dsRBD domain of HsDus2 is most likely bigger than expected (Figure 1A). To investigate whether the presence of HsDus2 substrates affects the proteolysis pattern, trypsinolysis was repeated in the presence of either NAD(P)H or bulk tRNA (Figure 1B). Increasing amounts of NADPH had clearly no effect, whereas tRNA addition enhanced the resistance of the dsRBD toward proteolysis. This protection of the dsRBD by the tRNA suggests that the dsRDB is implicated in tRNA binding. To examine the respective role of the two main HsDus2 domains, two constructions were made according to the boundaries established from the MALDI-TOF analysis (Supplementary Figure S2). The first construct, named HsDus2^dusD^, contains the Dus domain, whereas the second, designated HsDus2^dsRBD^, corresponds to the dsRBD. Both proteins were purified and characterized (see SI text). The UV-visible absorption spectrum of HsDus^dusD^ reporting the presence of flavin was not significantly different from that of full length *wild type* HsDus2, which indicates that the truncation has not altered the binding of the flavin cofactor (Supplementary Figure S3).

### Redox selectivity toward the hydride donor

The enzymatic reaction catalyzed by Dus is supposed to involve reduction of FMN by a reduced pyridine nucleotide, NADPH or NADH, followed by hydride transfer from reduced FMN hydroquinone to the uridine target to generate dihydrouridine (13). Oxidation of NADPH and NADH by menadione catalyzed by HsDus2, as a model reaction, was monitored under steady-state conditions. Although HsDus2 was able to catalyze both NADH and NADPH oxidation, the enzyme exhibited a clear preference for NADPH (Figure 1C). Indeed, a lower *K*_M_ value and a higher catalytic oxidation rate constant with NADPH as the hydride donor indicates a better catalytic selectivity for NADPH (ratio of *k*_cat_/*K*_M_^NADPH/NADH^ ∼ 6; Supplementary Table S1). HsDus2^dusD^ displayed the same preference for NADPH (Figure 1C and Supplementary Table S2). Addition of purified recombinant HsDus2^dsRBD^ in the activity test had no effect on NAD(P)H oxidation kinetics. Therefore, flavin redox-chemistry proceeds independently from the dsRBD.

### The Dus domain requires the dsRBD for dihydrouridine synthesis

The ability of HsDus2 to catalyze the formation of D was then assayed *in vitro* using, as the substrate, yeast bulk tRNAs prepared from a strain in which the dus2p gene had been knocked-out. This bulk tRNA contains ∼1.5 D, indicating the presence of D at positions other than position 20, as compared to ∼2.1 in bulk tRNA from the *wild type* strain (Figure 1D). Incubation of HsDus2 with this tRNA substrate restored full D content exclusively when NADPH was present in the reaction mixture (Figure 1D). In contrast, HsDus2 did not modify the D content of tRNA from the *wild type* strain. HsDus2^dusD^ could not catalyze D synthesis (Figure 1D). This result was puzzling since HsDus2^dusD^ contains both the catalytic and the putative tRNA-binding sites present in all Dus proteins. This clearly suggests that both domains are required for activity. No enzymatic activity was detected using an *in vitro* tRNA^Asp^ transcript substrate, suggesting that the activity relies on the presence of other modified bases in the tRNA substrate as reported in the case of yeast Dus2 (13).

### Full length HsDus2 but not its Dus domain complements a Δ*dus2* yeast strain by catalyzing formation of dihydrouridine 20

**Figure 2.**
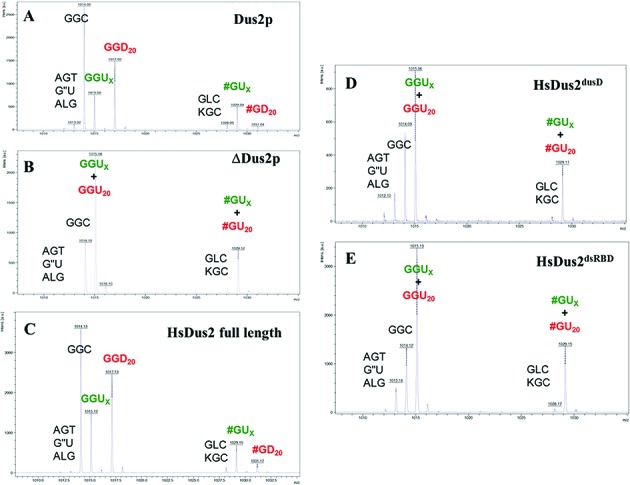
MALDI-MS analysis of digested tRNAs extracted from complemented Δdus2 yeast. MALDI-MS spectrum of RNAs fragments resulting from RNAse A digestion of bulk tRNAs originated from wild-type BY4741 (MATa; *his3*Δ 1; *leu2*Δ 0; *met15*Δ 0; *ura3*Δ 0) strain transformed by empty pCM190 vector (**A**), from Δ*dus2* BY4741 *dus2*::kanMX4 strain transformed by empty pCM190 (**B**) HsDus2-pCM190 (**C**) HsDus2^dusD^-pCM190 (**D**) or by HsDus2^dsRBD^-pCM190 vector (**E**). Peaks are identified by there *m/z* value and the corresponding trinucleotide obtained after RNAse A digest of bulk tRNA. T is ribothymidine, ′ is 1-methyladenosine, L is 2-methylguanosine, K is 1-methylguanosine and # is 2′-O-methylguanosine.

To confirm the *in vitro* assays and the importance of the dsRBD for activity we performed several yeast complementation tests with a BY4741 (MATa; *his3 Δ* 1; *leu2 Δ* 0; *met15 Δ* 0; *ura3 Δ* 0) *dus2*::kanMX4 strain. Bulk tRNA from the *wild type* strain was treated with RNase A and fragments of tRNAs containing the position 20 were analyzed by MALDI-MS. Among the 42 types of tRNAs expressed in yeast, 36 carry the D20. The fragments expected to contain D20 unambiguously are: GGD20 (*m/z* 1017.15) observed in 21 tRNAs and #GD20 (# = 2′-O-methylguanosine, *m/z* 1031.16) observed in 12 tRNAs. As shown in Figure 2A, the GGD20 and the #GD20 fragments are observed in tRNAs from the *wild type* yeast strain transformed with the empty pCM190 vector. The Δ*dus2* yeast strain transformed with the pCM190 vector exhibited a complete loss of both fragments (Figure 2B). This loss is strictly correlated with the gain of the GGU and #GU fragments. Complementation of the Δ*dus2* yeast strain with the pCM190-HsDus2 vector restored the GGD20 and the #GD20 content of the wild-type strain (Figure 2C). In contrast, when the Δ*dus2* yeast strain was transformed with the pCM190-HsDus2^dusD^ or pCM190-HsDus2^dsRBD^ plasmids, D20 was not synthesized (Figure 2D and E). These data demonstrate that a full-length HsDus2 is required for complementation of the Δ*dus2* yeast strain. To confirm these *in vivo* results and to ensure that the GGD20 and the #GD20 fragments are originated exclusively from the activity of HsDus2, the bulk tRNA from Δ*dus2* yeast strain was incubated in the presence of NADPH with either full-length HsDus2, HsDus2^dusD^ or HsDus2^dsRBD^. As shown in Supplementary Figure S5, the formation of these trinucleotide fragments are observed only in the presence of full length HsDus2 confirming that (i) the wild type enzyme catalyzes the formation of D20 both *in vivo* and *in vitro* and (ii) HsDus2^dusD^ is not catalytically competent for the reduction of U20, which strictly requires the HsDus2^dsRBD^.

### The dsRBD is the primary tRNA binding module

**Figure 3.**
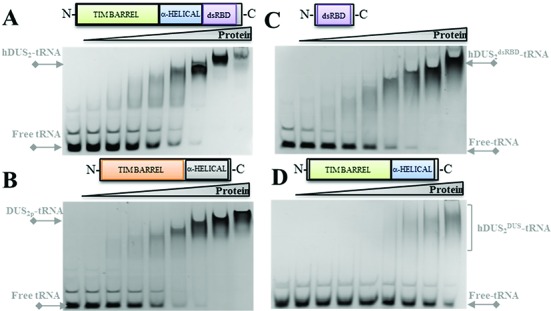
Implication of HsDus2 domains in the binding of tRNA. The ability of HsDus2 (**A**), *Saccharomyces cerevisiae* Dus2p (**B**), HsDus2^dsRBD^ (**C**), and HsDus2^dusD^ (**D**) to form stable complexes with bulk tRNA was tested by gel shift assays. The first line corresponds to the tRNA alone while the following lines correspond to the tRNA (10 μM) mixed with increasing amounts of protein (1.06, 2.12, 4.25, 8.5, 17, 34, 68 and 136 μM).

We observed that HsDus2^dusD^ did not co-purify with RNAs, contrasting with Dus2p, full-length HsDus2 and HsDus2^dsRBD^ that were all isolated as stable protein-RNA complexes when purified under low salt conditions (Supplementary Figure S4). This result was surprising since in HsDus2 orthologs, the Dus domain binds tRNA. To identify the tRNA binding module in HsDus2, the ability of each domain to form a stable protein–tRNA complex was evaluated by a gel shift assay using bulk tRNAs purified from a Δ*dus2* strain. Both HsDus2 and Dus2p formed a stable complex with tRNAs and exhibited similar apparent affinities (Figure 3A and B). Remarkably, HsDus2^dsRBD^ was also able to bind tRNA with an affinity in the same range of that of wild-type HsDus2 being ∼5–6 μM (Figure 3C and Supplementary Figure S6). In contrast, HsDus2^dusD^ was able to form a complex only at very high protein concentration revealing a much weaker affinity (∼15-fold) for the tRNA as compared to those of HsDus2 and HsDus2^dsRBD^ (Figure 3D and Supplementary Figure S6). HsDus2^dusD^ form a weak protein–tRNA complex but it is not catalytically competent. This failure of HsDus2^dusD^ to catalyze the formation of D20 might be due to a lack of productive tRNA orientation required for catalysis. This could be achieved by a cooperative action with the dsRBD that seems to play the central role in tRNA binding by HsDus2, a function that is normally assumed by the α-helical domain in Dus2p and in all bacterial Dus characterized so far. Although no activity was detected with a transcript, we observed that the full-length enzyme and its dsRBD were able to bind the *in vitro* tRNA^Asp^ transcript (Supplementary Figure S7). This suggests that prior post-transcriptional modifications are not required for tRNA binding but necessary for catalysis.

### Structure of HsDus2 catalytic domain

**Figure 4.**
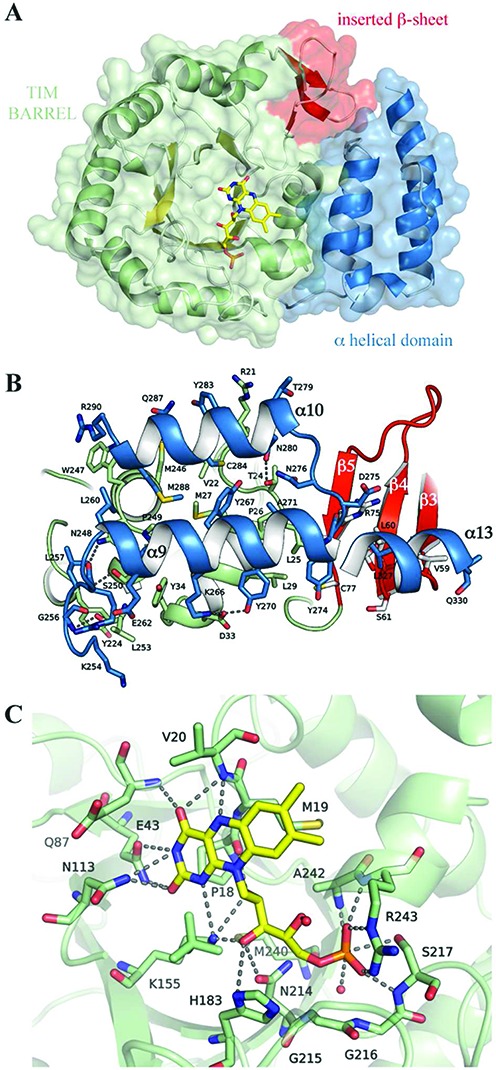
Structural characterization of the human Dus2 domain. (**A**) The structure of HsDus2^dusD^ shows the typical (α/β)_8_ TIM barrel fold (α helices in green, β strands in yellow) but contains three additional β strands (red). The FMN coenzyme (yellow) lies at the center of the barrel. This domain is directly connected to the α-helical sub-domain (blue). (**B**) Molecular interface between the TIM barrel and the helical sub-domain. The interface is mainly hydrophobic and stabilized by hydrogen bonds, π−cation interactions (R21-Y283, R290-W247) and one salt-bridge (D33-K276). For clarity, residues belonging to α11, α12 and part of α13 are not displayed. (**C**) HsDus2 active site showing the molecular interaction between the FMN redox cofactor (yellow) and the residues in its immediate vicinity (green). For simplicity, only the major FMN conformation is displayed.

**Figure 5.**
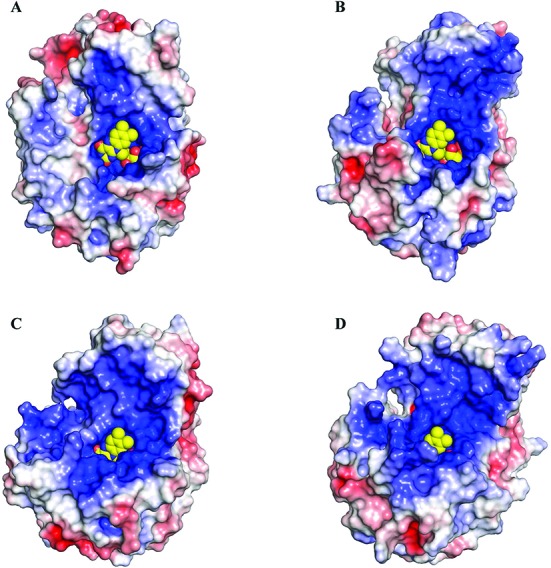
Comparison of the electrostatic surface of the TIM-barrel and helical domains of HsDus2 (**A**), DUS from *T*.*thermophilus* (**B**, pdb 3b0p), from *E*.*coli* (**C**, pdb 4bfa) and from a putative flavin oxidoreductase from *T*.*maritima* (**D**, pdb 1vhn). The electrostatic potentials displayed are within ± 5 kTe^−1^ and were calculated with APBS. The electrostatic surface of the helical domain of HsDus2 is less positively charged with respect to the bacterial dihydrouridine synthases and may explain the weak interaction of HsDus2^dusD^ with tRNA. The FMN is shown in yellow spheres.

HsDus2^dusD^ crystallized in the space group I222 with one molecule in the asymmetric unit.The structure confirmed the monomeric state of the domain observed by gel filtration (Supplementary Figure S4). The structure of HsDus2^dusD^ is organized into two distinct sub-domains (Figure 4A). The N-terminal region consists of a α8/β11 TIM barrel fold, in which a central barrel composed of eight parallel beta strands is surrounded by 8 alpha helices (Figure 4A and Supplementary Figure S8A). This structural arrangement is reminiscent of that found for flavoproteins of the glycolate oxidase family, as well as for dihydroorotate dehydrogenase and domain IV of dihydropyrimidine dehydrogenase, enzymes catalyzing comparable reactions (26). As expected for flavoenzymes with α/β barrels, a well-defined electron density, assigned to the redox FMN cofactor, lies at the center of the β barrel structure (Supplementary Figure S9A) close to the C-terminal end of barrel-forming strands with the *re*-face of the isoalloxazine ring oriented towards the barrel. An open groove running from the outer edge of the barrel to the *si*-face of the flavin facilitates the approach of the substrates (Figure 4A). Remarkably, the TIM barrel core diverges from the classical α8/β8 fold because three additional β strands inserted between Lys53 and Thr76 form a new antiparallel β sheet (β3−β5), a feature not observed in the other Dus structures (Supplementary Figure S10). This distinctive additional β sheet has not been predicted in the recent homology model (23). The TIM barrel is connected to the helical domain by a short 7-residue linker. The length of this linker varies among the Dus family. The helical sub-domain is formed by a 5-helix bundle that caps the top of the FMN binding site, while it is composed of four helices in the other known Dus structures (14–16). A consequence of this additional helix is that the C-terminus borne by helix α13 points toward the opposite direction compared to the other Dus, thereby orienting the dsRDB domain. Besides these structural divergences, both the TIM barrel and helical sub-domains are present among Dus enzymes and constitute the bona-fide ‘Dus catalytic domain’ (Supplementary Figure S10). These two sub-domains share an interaction surface of ∼ 1168 Å^2^ (27), which is mainly hydrophobic but stabilized by additional electrostatic contacts, notably hydrogen bonds and π−cation interactions (Figure 4B). The aromatic residues present in the interface participate in the orientation of the helical sub-domain. The relative orientation of the two sub-domains is known to differ among Dus (15). A structural alignment of human, *E. coli* and *T. thermophilus* Dus domains confirmed this distinctive feature among the Dus protein (Supplementary Figure S11). In HsDus2, the interaction area between these sub-domains is slightly larger than that of the bacterial Dus (varying between 868 and 1017 Å^2^) due to the presence of the inserted (β3−β5) sheet which accounts for ∼15% of the surface. This β-sheet fills a large gap between the TIM barrel and the C-terminal end of the helical sub-domain and interacts with the additional helix of the helical sub-domain (Figure 4A). Remarkably, this domain displays a less positively charged surface of the helical sub-domain of HsDus2 compared to its known Dus counterparts (Figure 5), which could explain why HsDus2^dusD^ exhibit a weak tRNA binding.

### Structure of the FMN-binding site

The structure of the FMN binding site is shown in Figure 4C (and Supplementary Figure S9). The FMN cofactor lies inside a deep positively charged crevice, stabilizing the negative charge of the phosphate moiety of the cofactor (Figure 4C). All the constituting parts of the FMN, i.e the isoalloxazine ring and the ribityl phosphate chain make extensive interactions with surrounding amino acids residues, ensuring a tight binding and a proper orientation of the entire coenzyme. The dimethyl benzene ring of FMN is bound to the protein by hydrophobic stacking interactions with the side chains of the widely conserved Met19 (Figure 4C and Supplementary Figure S9). The Sδ of Met19 is in van der Waals contact (∼4.5 Ǻ) with the C8 methyl group of the isoalloxazine ring. The side chains of three extremely conserved residues, Gln87, Asn113 and Lys155 interact with the pyrimidine ring of FMN, thereby stabilizing the negative density delocalized over the N1–C2 and the C2–O2 bonds. The Nϵ atom of Lys155 assumes a double function: located at 3.5 Ǻ from the N1 atom of FMN, it neutralizes the building negative charge on the isoalloxazine ring but it also interacts with the C3′ hydroxyl group of the ribityl moiety. Gln87 interacts with both O2 and N3 of FMNH^−^, while O4 is hydrogen bonded with the NH amide bond of the non-conserved residues Glu43 and Val20. The ribityl moiety of FMN interacts with the protein essentially throughout its C3′-OH group.

HsDus2^dusD^ binds the FMN cofactor non-covalently at the carboxy-terminal end of the β-barrel, above β-strands 1 and 8 (Figure 4A and Supplementary Figure S9). The isoalloxazine ring occupies a similar orientation as in *Tth*Dus, *Tm*Dus and DusC with the redox reactive N5 atom oriented towards the solvent, ready to receive the hydride from NADPH (Figure 4C). In contrast to other structurally characterized Dus proteins, in which the isoalloxazine ring is planar (14–16), the electron density of the FMN cofactor in HsDus2^dusD^ revealed a ‘butterfly’ bending at the N(5)–N(10) hinge, with the concave side corresponding to the *re*-face of the ring. Since the oxidized flavin ring system is widely described to be planar (28,29), while bending along the N(5)–N(10) axis occurs only upon reduction of the isoalloxazine ring, the FMN cofactor of HsDus2^dusD^ was likely reduced by the X-ray beam during data collection (30). The positive charge carried by the side chain of Lys155 points toward the N1 of the isoalloxazine, consistent with FMN carrying a negative charge on N1 and therefore being in the FMNH^−^ state. Interestingly, the ribityl-phosphate chain adopts two slightly different conformations compatible with the electron density map that are stabilized by similar interactions (Supplementary Figure S9).

Like in most Dus structures, a 13-amino-acid loop spanning residues 116 to 128 and located near the active site and inserted in the TIM barrel between β6 and the small α5 helix was disordered. This loop contains the highly conserved Cys110, which has been proposed to function as a proton exchange site (13,16). The corresponding loop gets ordered in the presence of tRNA (16,17).

### Structure of the dsRBD: a domain with potential binding for both tRNA and protein partners

**Figure 6.**
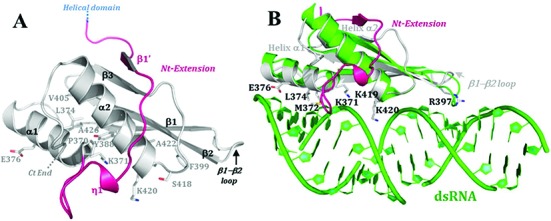
Structure of the dsRBD and model of dsRNA binding mode. (**A**) Crystal structure of HsDus2^dsRBD^ (Glu347-Lys451) showing all the residues conserved among the dsRBDs family and the novel extension at the N-terminal region of the canonical αβββα fold. (**B**) Structural alignment of HsDus2^dsRBD^ (in gray) with *X. leavis* RBPA dsRBD (pdb 1DI2) in complex with a dsRNA fragment (in green). The conserved residues known to be implicated in the dsRNA binding in classical dsRBDs are shown in HsDus2^dsRBD^. Both dsRBDs align with the exception of the β3 strand and the β1−β2 loop, which are oriented in the opposite direction from dsRNA. In this superposition, several conserved regions of HsDus2^dsRBD^ usually involved in RNA binding in dsRBDs are in contact with the dsRNA without provoking clashes. Therefore, this model could reflect the way HsDus2^dsRBD^ binds a double stranded region of its tRNA substrate. Helix α1 snugly fills the minor groove of the dsRNA, while the N-terminal tip of helix α2 caps the major groove. The basic residues Lys371, Lys419 and Lys420, located in conserved motifs, are oriented towards the phosphodiester backbone of nucleotides from the major groove as in the functional conformations observed in RNAse III (34). Moreover, Arg397 located in the N-terminal region of the β2 strand contacts the minor groove of the dsRNA by interacting with a nucleotide ribose moiety, contrasting with the interaction made by the β1−β2 loop in the RNA binding protein A complex. Although the N-terminal extension carries several basic residues, these do not interact with the dsRNA.

Overall, the dsRBD structure contains an N-terminal extension (from Ser350 to residue 368) and the canonical αβββα topology (from Thr369 to Gly435) (Figure 6A and Supplementary Figure S12A). The N-terminal extension wraps the entire dsRBD (Figure 6A and Supplementary Figure S12B), thus leading to a globular shape of the domain. This extension is made of a short β strand (β1) that interacts with the βββ fold, thereby forming an extended β-sheet followed by a long unstructured linker and a short α−helical turn (η1) (Figure 6A). A few other dsRBDs have been shown to bear an extension at either the N- or C-terminus (Supplementary Figure S12B). These extensions are often implicated in protein-protein interaction (31,32). In HsDus2, this extension may serve the same purpose.

The helices of the αβββα canonical fold pack against three antiparallel β strands as generally observed in classical dsRBDs such as in Xrpa (33). However, some variations are observed, notably a shorter β3 sheet and β1−β2 loop (Figure 6). Besides interacting with dsRNA, this loop in dsRBDs has also been proposed to bind proteins (34–38). Several hydrophobic interactions lying at the interface between the α helices and β sheet stabilize the canonical fold (Figure 6A). In particular, these interactions involve the conserved residues Tyr388 and Phe399 (Supplementary Figure S12A) which are known to participate in dsRNA binding by maintaining optimal orientation of key positively charged residues (34,38). Lys371, Lys419 and Lys420, located in conserved motifs as well as Arg397 are conserved basic residues among the dsRBDs and they are known to participate in the interaction with the dsRNA (Figure 6 and Supplementary Figure S12).

To illustrate how the dsRBD of HsDus2 might bind its tRNA substrate which contains some dsRNA regions, a structural alignment of HsDus2^dsRBD^ with the dsRBD of *Xenopus leavis* RNA binding protein A in complex with dsRNA was achieved. The low r.m.s.d.value (3.29 Ǻ on 61 Cα of the canonical fold) validates the relative orientation of the dsRNA with respect to the protein. In the resulting model (Figure 6B) the dsRNA interacts with the protein via a ∼14 nucleotides-fragment, which can also be seen as the double-stranded region of the tRNA substrate corresponding to the acceptor and T-psi arm. The model also shows that the N-terminal extension does not interact with RNA suggesting its availability for protein-protein interaction.

## CONCLUSION

**Figure 7.**
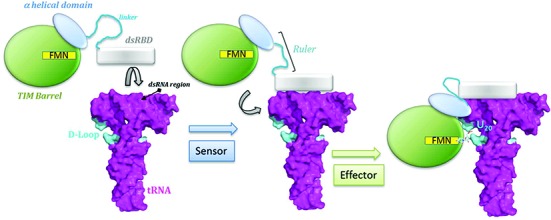
Proposed mechanism for tRNA binding. The primarily binding of the dsRBD at the anticodon arm considered as a ‘reference point’ enables to measure the distance to the modification site. Then, the recruitment of the other modules via a flexible linker between the dsRBD and the helical domain operating as a molecular ruler enables the fine-tuned positioning of the catalytic domain around the tRNA elbow. Finally, the catalytic domain flips out U20 from its initial position to the FMN binding pocket for catalysis.

Using biochemical analysis and structural characterization, we here show that HsDus2 uses a unique strategy for binding and modifying its tRNA substrate. In contrast to other Dus proteins, HsDus2 has a dsRBD, which is the primary tRNA binding site. This interaction is essential for bringing the Dus domain in close vicinity of the D-loop of tRNA, allowing the FMN cofactor to transfer a hydride to uridine (Figure 7). HsDus2 thus employs a new tRNA recognition mode among tRNA-modifying enzymes that target loops within the tRNA. The reason why HsDus2 evolved to contain a dsRBD for a cooperative binding of tRNA is unclear. However, it might be related to the dual role of HsDus2 in tRNA metabolism and translational factor regulation mediated via protein/protein contacts. By interacting with both the substrate and regulatory proteins, the dsRBD would be the key effector of HsDus2. This working hypothesis is under investigation.

Note that during the course of revision of our manuscript, a paper reporting the structure of HsDus2^dusD^ has been published (39).

## ACCESSION NUMBERS

The atomic coordinates have been deposited in the Protein Data Bank (www.pdb.org) with the following PDB ID codes: 4WFS (catalytic domain) and 4WFT (dsRBD domain).

## Supplementary Material

SUPPLEMENTARY DATA
