# Supplementary material for: An extended dsRBD is required for post-transcriptional modification in human tRNAs
Source: Nucleic Acids Res. 2015 Oct 1;43(19):9446–56. doi: 10.1093/nar/gkv989 (PMC4627097; doi:10.1093/nar/gkv989)
Supplement: SUPPLEMENTARY DATA [file supp_43_19_9446__index.html]

An extended dsRBD is required for post-transcriptional modification in human tRNAs — An extended dsRBD is required for post-transcriptional modification in human tRNAs — SUPPLEMENTARY DATA 

# An extended dsRBD is required for post-transcriptional modification in human tRNAs

## SUPPLEMENTARY DATA

- SUPPLEMENTARY DATA
