# Supplementary material for: An extended dsRBD is required for post-transcriptional modification in human tRNAs
Source: Nucleic Acids Res. 2015 Oct 1;43(19):9446–56. doi: 10.1093/nar/gkv989 (PMC4627097; doi:10.1093/nar/gkv989)
Supplement: SUPPLEMENTARY DATA [file supp_gkv989_nar-01457-r-2015-File002.docx]

**Supplementary information**

**SI Materials and Methods**

**Cloning of Dus2p, HsDus2 and its subdomains**

The plasmid pEFX-04 containing the ORF encoding for N-terminal histidine tagged Dus2p was a generous gift from Professor Eric M. Phizicky (University of Rochester). The HsDus2 full length ORF cloned between NotI and BamHI in pCDNA3.1 was a generous gift from Professor Rekha C. Patel (University of South Carolina ref). The Amplified *HsDus2* gene was cloned into the expression vector pET11d between the BamHI and NC0I sites. The full length HsDus2 carries a (His)_6_-tag at the N-terminus. Three different constructions of HsDus2 domains have been designed according to the mild proteolysis pattern of the full length protein (figure 1A&B). These constructs have also been cloned in pET11d between the BamHI and NC0I sites. The first construct carries an (His)_6_-tag at the N-terminal region and encodes the HsDus2 sequence from Met1 to Arg333, including both the α/β barrel and α-helical domains. The second construct is identical to the first one except that the 12 first amino acids (Met1-Lys13) were truncated. Finally, the third construct encodes the region Thr338 to Lys450 which corresponds to the dsRBD with a (His)_6_-tag was placed at the C-terminus. A construct of the dsRBD containing the last 43 C-terminal residues of HsDus2 was not considered because this region was predicted as disordered by bioinformatics ([1](#_ENREF_1)) and thus could be refractory to crystallization.

**Expression and purification of Dus2p, HsDus2 and its subdomains**

All the recombinant proteins were expressed and purified according the same procedure. The recombinant proteins were overexpressed in *E. coli* BL21 (DE3) Star Codon Plus. The proteins expressions were performed in LB medium supplemented with 220 µM riboflavin and 100 µg/mL ampicillin. Cell growth was carried out at 37°C until the absorbance at 600 nm reached ~ 2; then protein expression was induced with 0.5 mM isopropyl-1-thio-β-D-galactopyranoside (IPTG). After overnight growth at 29°C, cells were harvested by centrifugation at 5500 g for 15 minutes at 4°C. The pellets were suspended in 50 mM Na_2_HPO_4_ pH 8, 150 mM NaCl, 25 mM imidazole and 10% glycerol (Buffer A) containing 10 mM β-mercaptoethanol and 1 tablet of protein inhibitors cOmplete, EDTA-free (Roche). The cells were lysed by sonication and the lysate was clarified by centrifugation at 13000 rpm at 4°C for 70 min. The resulting supernatant was loaded onto a Ni^2+^-NTA agarose affinity column (Qiagen) pre-equilibrated with buffer A. After washing with 2 column volumes of buffer A, elution was carried out with 50 mM Na_2_HPO_4_ pH 8, 300 mM NaCl, 250 mM imidazole and 10% glycerol (buffer B). Protein concentration was determined by the Bradford method and the samples were concentrated using a 20 mL Vivaspin centrifugal concentrator (10 000 MWCO PES membrane; Sartorius). The presence of RNA copurified with the protein was checked by recording the UV-visible absorption of the sample. To remove *E. coli* RNA contaminant bound to the proteins, purification by Ni^2+^-NTA agarose affinity chromatography was performed using a buffer containing 50 mM Na_2_HPO_4_ pH 8, 2 M NaCl, 25 mM imidazole and 10% glycerol (buffer C). To further purify the samples, the proteins were loaded on a Superdex S200 10/300 GL or S200 16/600 column (GE healthcare Inc.) equilibrated with 25 mM Tris pH 8 and 150 mM NaCl and the elution was followed by recording simultaneously three wavelengths (260 nm for nucleic acids, 280 nm for proteins and 450 nm for the flavin cofactor). The fractions containing the desired protein were analyzed by SDS-PAGE and pooled before their storage at -80°C.

**Preparation of bulk and *in vitro* tRNA transcripts**

Bulk tRNA was extracted from yeast strains BY4741 (MATa; his3Δ 1; leu2Δ 0; met15Δ 0; ura3Δ 0) and its derivative ∆dus2 derivative (MATa; his3Δ 1; leu2Δ 0; met15Δ 0; ura3Δ 0; YNR015w::kanMX4). Cells were grown in 500 ml YPD (peptone 2%; yeast extract 1% and glucose 2%) to an OD600 of 0.8. Pelleted cells were washed twice in 20 ml TMN (10 mM Tris-HCl (pH 7.5), 10 mM MgCl2, 0.15 M NaCl). The final pellet was resuspended in 40 ml TMN: acidic phenol (pH 4.5) (1:1 ratio) and incubated for 20 minutes at room temperature on a rotating wheel. After centrifugation, the aqueous phase was supplemented with 0.8 M LiCl and incubated overnight at 4°C to precipitate high molecular mass molecules. The precipitate was eliminated by centrifugation and the supernatant was supplemented with two volumes of 100% ethanol and incubated at -20°C for 2 hours to precipitate tRNAs. After centrifugation, pelleted tRNAs were washed twice in 70% ethanol and resuspended in 1 ml of RNAse-free water. tRNAs were further purified using the NucleoBond RNA/DNA 400 kit (Macherey-Nagel) following manufacturer's instructions, except that the elution step was performed with 5 ml of 100 mM Tris-acetate (pH 6.3); 15% ethanol and 600 mM KCl. In vitro transcription of yeast tRNA^Asp^ was performed using an oligonucleotide template containing ribose 2′-methoxy modifications at the last 2 5′ terminus nt ([2](#_ENREF_2)) and purified by electrophoresis on a 12% polyacrylamide, 8 M urea gel. The band containing RNA was located by UV-shadowing and excised from the gel. RNA was extracted with 0.3 M sodium acetate pH 5.2 and loaded onto a HiTrap DEAE Sepharose column (GE Healthcare Inc.) pre-equilibrated in the same buffer. After elution with 3 M sodium acetate pH 5.2, the transcript was ethanol precipitated and desalted on a PD-10 column (Sephadex-G25 medium; GE Healthcare Inc).

**Identification of HsDus2 domains**

To identify HsDus2 domains, mild trypsinolysis was carried out at room temperature in 50 mM HEPES pH 7.5, 50 mM NaCl and 5% glycerol in a final volume of 20 µl containing 8.8 µM of recombinant HsDus2 and 338 nM of trypsin. Several conditions were tested with or without NADPH (100 µM, 500 µM and 1 mM) and bulk tRNA (2.97 µM). The trypsinolysis was stopped by adding 40 nM of Pefabloc® and the proteolysis patterns were analyzed on SDS-PAGE. To determine whether the domains were soluble the products of mild proteolysis were loaded on a Ni-NTA column. The N-terminal domain, retained on the affinity column, was eluted. The domain was soluble and contained the flavin cofactor, confirming the presence of an intact catalytic site.

To identify the boundaries of the domains obtained from the mild proteolysis experiment, MALDI mass spectrometry analysis was performed according to an established protocol ([3](#_ENREF_3)) after total enzymatic digestion of the excised bands. Protease-generated peptide mixtures were analyzed by MALDI-TOF (Voyager-DE STR, Applied Biosystems). Mass spectrometry measurements were carried out at a maximum accelerating potential of 20 kV, in the positive reflectron mode. Peak lists were generated by the Data Explorer software (Applied Biosystems), and processed data were submitted to the FindPep software (available on the World Wide Web) using the following parameters: protein sequence, HsDus2; mass tolerance, 50 ppm; digest reagents, trypsin or AspN/N-terminal Glu or none; fixed modification, carbamidomethylation of cysteines; possible modification, oxidation of methionines.

**Dihydrouridine assay**

In vitro activity tests were performed by incubating for 2 hours at 30°C 60 µM tRNAs, 20 µM protein and 1 mM NADPH in 50 mM Tris pH 7.5, 100 mM ammonium acetate, 10 mM MgCl_2_, 2 mM DTT and 15% glycerol (buffer D). tRNAs were separated after phenol extraction with 50 µL of phenol/chloroform (1:1) and subsequent centrifugation at 13000 rpm at room temperature for 10 minutes. Then 10 µL of 3 M sodium acetate and 250 µL of 96% ethanol were added to the aqueous phase and this mixture was incubated for 1 hour at -80°C. The tRNAs were pelleted by centrifugation at 13000 rpm for 20 minutes at 4°C and the ethanol was removed from the sample. Then the tRNAs were washed with 200 µL of 70% ethanol and centrifuged again for 5 minutes at 4°C. After drying, tRNAs were suspended in 50 µL water and its concentration was assessed by monitoring its absorbance at 260 nm. The dihydrouridine content was determined according to a previously established colorimetric method based on the quantification of the acyclic ureido group formed by alkaline cleavage of the dihydrouridine ring. Briefly, 5 µL of 1 M KOH was added and the mixture was incubated for 30 minutes at 40°C. The solutions were neutralized by adding 25 µL of concentrated H_2_SO_4_ followed by 25 µL of a 3% solution in 2,3-butanedione monoxime (Sigma Aldrich) and 25µL of a saturated solution in N-Phenyl-p-phenylenediamine (Sigma Aldrich). Samples were then heated at 95°C for 10 minutes and cooled to 55°C. Following addition of 50 µL of 1 mM FeCl_3_ in concentrated H_2_SO_4_, a violet-red coloration appeared. The absorbance measured at 550 nm allowed the quantification of dihydrouridine according to a calibration curve based on concentrations of dihydrouracil ranging from 10 to 150 µM.

**Crystallization, data collection and structure determination**

Crystals of both HsDus2 domains were obtained at 292 K using the hanging drop vapor diffusion method. Crystals of HsDus2^dusD^ were obtained after mixing 1 µl of protein at 8 mg/ml in 25 mM Tris pH 8.0, 150 mM NaCl with 1 µL of reservoir solution (2.2 M ammonium sulfate, 2% v/v isopropanol). HsDus2^dsRBD^ at 20 mg/mL crystallized after it was mixed with an equal volume of 100 mM HEPES pH 6.5, 100 mM sodium acetate and 30% PEG 2000 MME. Crystals were cryoprotected with a reservoir solution supplemented with 20% (v/v) of glycerol then flash frozen in liquid N_2_. X-ray diffraction data were collected on a single crystal at 100 K on beamline PROXIMA-I at the SOLEIL synchrotron (Saint-Aubin, France). Data were indexed, processed, and scaled with XDS ([4](#_ENREF_4)). Data collection and processing statistics are given in Table S1.

Crystals of HsDus2^dusD^ (residues 14 to 330) diffracting up to 2.68 Å resolution were orthorhombic (space group I222) and contained an estimated solvent content of 60% (Vm=3.06), corresponding to one molecule per asymmetric unit. Initial structure solution of HsDus2^dusD^ was obtained using the MR-ROSETTA procedure ([5](#_ENREF_5)) within PHENIX 1.8.4.1496 (ROSETTA 3.5 ([6](#_ENREF_6))), starting with a sequence alignment file obtained from HHpred ([7](#_ENREF_7)). The molecular replacement solution was found using *T. thermophilus* DusC (PDB code 3B0P) as template (19% sequence identity). The whole procedure resulted in a model containing residues 25-57, 83-115, 132-185, 198-216, 236-320 with R_work_/R_free_ of 0.32/0.36. Interpretable unfilled map for FMN and protein chain was subjected to automated building with Buccaneer 1.5.2 ([8](#_ENREF_8)). This step gave a new model that was 91% complete with R_work_/R_free_ of 0.27/0.30 and overall FOM of 0.81. Several steps of manual building in Coot ([9](#_ENREF_9)) and refinement in Buster 2.10 ([10](#_ENREF_10)) led to a final model with the following missing residues (sequence numbering according to wt: 116-128 and 331-333 as well as the first five residues of the histidine-tag). Refinement statistics are given in Table S1.

The dsRBD domain crystallized at 1.7 Å in the P32 group space with three molecules in the asymmetric unit. All the copies are similar, as evidenced by low rmsd obtained from their structural alignment (mol A vs mol B ~ 0.12 Å for 72 Cα and mol A vs mol C ~ 0.18 Å for 78 Cα). The electron density corresponding to the N- (Thr339-Glu347) and C- (Glu443-Lys451) terminal regions was not visible, suggesting highly flexible regions (Table S1). Regarding the resolution of the HsDus2^dsRBD^ structure, the SAD-phasing method using sulfur atom anomalous dispersion at remote wavelengths, was used. The sulfur-SAD dataset was collected on beam line Proxima-1 (Soleil). The wavelength was set to 1.8 Å and 3 datasets were collected on a single cristal with a 3 circle axis –goniostat at κ angles 0,15,-20° with 0.2° oscillation range on the φ axis. A data set collected at 0.98 Å was used as reference for scaling and merging. Data were integrated, scaled and merged with XDS/XSCALE to obtain a highly redundant dataset (multiplicity of 21.3).

The substructures (15 sulfur atoms per asymmetric unit) were solved with *SHELXD* using 1000 trials and *SHELXE* was used to attest for successful phasing ([11](#_ENREF_11)). A cut-off resolution of 2.9 Å was used for the heavy atom search. The substructures were refined and completed with *PHASER* ([12](#_ENREF_12)) using all data. Phases for both enantiomorphs were generated and were subjected to automatic density modification as implemented in *Parrot* ([13](#_ENREF_13)) to improve phases and break phase ambiguity. *Buccaneer* ([8](#_ENREF_8)) was used for automated model building. The model was refined by alterning manual building in Coot ([9](#_ENREF_9)) and refinement in REFMAC ([14](#_ENREF_14)). This model was further refined using a dataset collected to 1.7 Å resolution on another cristal. The final model of HsDus2^dsRBD^ was obtained after several manual building and refinement cycles using PHENIX Refine ([15](#_ENREF_15)) and Coot. Refinement statistics are summarized in Table S1.

**SI Results and Discussion**

**Recombinants Dus2p, HsDus2 and HsDus2^dsRBD^ are isolated with RNAs from *E. coli*.~~-~~**The recombinant human and yeast enzymes as well as the human dsRBD contained RNAs after the nickel affinity column. In the UV-visible absorption spectrum of HsDus2, HsDus2^dsRBD^ and Dus2p (Figure S3), the presence of a band of high intensity at 260 nm together with a broad band at ~340 nm attributed to 4-thiouridine (base modification found exclusively in tRNA of eubacteria) suggested that these proteins had been isolated with *E. coli* tRNAs. Similar results were observed when recombinant dihydrouridine synthases from *Thermus thermophilus* (*Tth*Dus) and from *Saccharomyces cerevisiae* (Dus2p) were expressed in *E. coli* ([16](#_ENREF_16), [17](#_ENREF_17)). The nature of the RNAs isolated with recombinant *Tth*Dus was confirmed to be tRNAs by mass spectrometry ([17](#_ENREF_17)). To remove all traces of RNAs from the proteins, an extensive washing procedure with high ionic strength (2 M NaCl) of the protein/RNA complex was performed on the nickel affinity column. The UV-visible spectrum of the resulting RNA-free HsDus2 and Dus2p retained all the characteristic bands of the flavin but not those attributed to RNAs (figure S3). The same procedure was repeated with HsDus2^dsRBD^ and, as evidenced by the UV-visible spectrum, the RNAs were completely removed from the protein (figure S3). These results suggest that HsDus2, HsDus2^dsRBD^ and Dus2p were able to form non covalent RNA/protein complexes with *E. coli* RNAs, likely tRNAs. In contrast, HsDus2^dusD^ was isolated from *E. coli* without any traces of RNAs, even at low ionic strength (figure S3), suggesting its weak affinity or its inability to interact with tRNAs. HsDus2^dusD^ retained the ability to bind the flavin cofactor, as evidenced by absorbance bands at 365 and 455 nm attributed to the oxidized hydroquinone (figure S3C).

The homogeneity of the proteins and RNA/protein complexes isolated from *E. coli* was analyzed by size exclusion chromatography. In absence of RNAs, full length HsDus2, HsDus2^dusD^ HsDus2^dsRBD^ and Dus2p eluted at volumes expected for monomeric proteins. The elution profiles indicated homogenous proteins and the absorbance at 280 nm higher than that at 260 nm confirmed the absence of RNA (figure S4, high salt). In contrast, the elution volumes corresponding to the proteins co-purified with *E coli* RNAs were smaller than those observed with RNA-free proteins, which indicated stable complexes (figure S4, low salt). According to the mean size of tRNAs (~ 24 kDa) and the elution volumes of the RNA/protein complexes, we can propose that one molecule of HsDus2^dsRBD^ binds to one tRNA molecule (figure S4), while this is less clear for HsDus2 and Dus2p, probably due to a less globular shape influencing the elution profile.

Figure S1. Sequences alignment of HsDus2^dusD^ with several bacterial Dus proteins.

Figure S2. MALDI analysis of polypeptides originated from mild trypsinolysis. The first data set was obtained by fully digesting the intense band at 38 kDa, while the second data set was obtained from the full digestion of the weak band at 14 kDa (Fig. 1B). Matching peptides are shown in red. Sequence coverage of 53% (for the Dus domain) and 32% (for the dsRBD) were obtained respectively for the first and the second data set when compared with the sequence of wild type HsDus2. Note that below the numbering of the residues starts at the first histidine of the Nt-His tag while in the main text of the manuscript it starts at the Nt-Methionine.

1 HHHHHHmiln slslcyhnkl ilapmvrvgt lpmrllaldy gadivyceel

51 idlkmiqckr vvnevlstvd fvapddrvvf rtcereqnrv vfqmgtsdae

101 ralavarlve ndvagidvnm gcpkqystkg gmgaallsdp dkiekilstl

151 vkgtrrpvtc kirilpsled tlslvkrier tgiaaiavhg rkreerpqhp

201 vscevikaia dtlsipvian ggshdhiqqy sdiedfrqat aassvmvara

251 amwnpsiflk eglrpleevm qkyiryavqy dnhytntkyc lcqmlreqle

301 spqgrllhaa qssreiceaf glgafyeett qeldaqqarl saktseqtge

351 paedtsgvik mavkfdrray paqitpkmcl lewcrrekla qpvyetvqrp

401 ldrlfssivt vaeqkyqstl wdkskklaeq aaaivclrsq glpegrlgee

451 spslhkrkre apdqdpggpr aqelaqpgdl ckkpfvalgs geesplegw

| Start - End | Observed | Mr (expt) | Mr (Calc) | ppm | Miss | Sequence |
| --- | --- | --- | --- | --- | --- | --- |
| 20 – 27 | 912.5600 | 911.5527 | 911.5626 | -11 | 0 | K.LILAPMVR.V |
| 20 – 27 | 928.5509 | 927.5436 | 927.5575 | -15 | 0 | K.LILAPMVR.V Oxidation (M) |
| 28 – 34 | 773.4576 | 772.4504 | 772.4265 | 31 | 0 | R.VGTLPMR.L |
| 35 – 54 | 2326.2245 | 2325.2173 | 2325.1814 | 15 | 0 | R.LLALDYGADIVYCEELIDLK.M |
| 60 – 77 | 2031.1140 | 2030.1067 | 2030.0433 | 31 | 1 | K.RVVNEVLSTVDFVAPDDR.V |
| 61 – 77 | 1875.0157 | 1874.0084 | 1873.9422 | 35 | 0 | R.VVNEVLSTVDFVAPDDR.V |
| 90 – 101 | 1339.6700 | 1338.6628 | 1338.6238 | 29 | 0 | R.VVFQMGTSDAER.A |
| 90 – 101 | 1355.6658 | 1354.6586 | 1354.6187 | 29 | 0 | R.VVFQMGTSDAER.A Oxidation (M) |
| 108 – 124 | 1830.9579 | 1829.9506 | 1829.8652 | 47 | 0 | R.LVENDVAGIDVNMGCPK.Q |
| 130 – 145 | 1601.8860 | 1600.8787 | 1600.8130 | 41 | 1 | K.GGMGAALLSDPDKIEK.I |
| 164- 176 | 1427.8871 | 1426.8799 | 1426.8283 | 36 | 0 | R.ILPSLEDTLSLVK.R |
| 181 – 191 | 1065.6280 | 1064.6207 | 1064.6091 | 11 | 0 | R.TGIAAIAVHGR.K |
| 194 – 207 | 1707.9097 | 1706.9024 | 1706.8410 | 36 | 1 | R.EERPQHPVSCEVIK.A |
| 208 – 237 | 3282.5738 | 3281.5665 | 3281.5953 | -9 | 0 | K.AIADTLSIPVIANGGSHDHIQQYSDIEDFR.Q |
| 238 – 249 | 1191.6424 | 1190.6351 | 1190.6077 | 23 | 0 | R.QATAASSVMVAR.A |
| 250 – 260 | 1277.6985 | 1276.6912 | 1276.6638 | 21 | 0 | R.AAMWNPSIFLK.E |
| 250 – 260 | 1293.6965 | 1292.6893 | 1292.6587 | 24 | 0 | R.AAMWNPSIFLK.E Oxidation (M) |
| 261 – 272 | 1444.7944 | 1443.7871 | 1443.7391 | 33 | 1 | K.EGLRPLEEVMQK.Y Oxidation (M) |
| 261 – 275 | 1860.9895 | 1859.9822 | 1859.9927 | -6 | 2 | K.EGLRPLEEVMQKYIR.Y |
| 276 – 288 | 1616.7899 | 1615.7826 | 1615.7267 | 35 | 0 | R.YAVQYDNHYTNTK.Y |
| 289 – 296 | 1143.5333 | 1142.5260 | 1142.5035 | 20 | 0 | K.YCLCQMLR.E |
| 289 - 296 | 1159.5404 | 1158.5332 | 1158.4984 | 30 | 0 | K.YCLCQMLR.E Oxidation (M) |
| 297 – 305 | 1043.5211 | 1042.5138 | 1042.5043 | 9 | 0 | R.EQLESPQGR.L |
| 306 – 314 | 982.5447 | 981.5374 | 981.5356 | 2 | 0 | R.LLHAAQSSR.E |
| 315 -339 | 2876.2900 | 2875.2827 | 2875.2970 | -5 | 0 | R.EICEAFGLGAFYEETTQELDAQQAR.L |

1 HHHHHHmiln slslcyhnkl ilapmvrvgt lpmrllaldy gadivyceel

51 idlkmiqckr vvnevlstvd fvapddrvvf rtcereqnrv vfqmgtsdae

101 ralavarlve ndvagidvnm gcpkqystkg gmgaallsdp dkiekilstl

151 vkgtrrpvtc kirilpsled tlslvkrier tgiaaiavhg rkreerpqhp

201 vscevikaia dtlsipvian ggshdhiqqy sdiedfrqat aassvmvara

251 amwnpsiflk eglrpleevm qkyiryavqy dnhytntkyc lcqmlreqle

301 spqgrllhaa qssreiceaf glgafyeett qeldaqqarl saktseqtge

351 paedtsgvik mavkfdrray paqitpkmcl lewcrrekla qpvyetvqrp

401 ldrlfssivt vaeqkyqstl wdkskklaeq aaaivclrsq glpegrlgee

451 spslhkrkre apdqdpggpr aqelaqpgdl ckkpfvalgs geesplegw

| Start – End | Observed | Mr (expt) | Mr (Calc) | ppm | Miss | Sequence |
| --- | --- | --- | --- | --- | --- | --- |
| 20 – 27 | 912.5765 | 911.5692 | 911.5626 | 7 | 0 | K.LILAPMVR.V |
| 20 – 27 | 928.5697 | 927.5624 | 927.5575 | 5 | 0 | K.LILAPMVR.V Oxidation (M) |
| 28 – 34 | 773.4208 | 772.4136 | 772.4265 | -17 | 0 | R.VGTLPMR.L |
| 28 – 34 | 789.4129 | 788.4056 | 788.4215 | -20 | 0 | R.VGTLPMR.L Oxidation (M) |
| 61 – 77 | 1874.9689 | 1873.9616 | 1873.9422 | 10 | 0 | R.VVNEVLSTVDFVAPDDR.V |
| 90 – 101 | 1339.665 | 1338.6582 | 1338.6238 | 26 | 0 | R.VVFQMGTSDAER.A |
| 90 – 101 | 1355.6574 | 1354.6502 | 1354.6187 | 23 | 0 | R.VVFQMGTSDAER.A Oxidation (M) |
| 108 – 124 | 1830.8994 | 1829.8921 | 1829.8652 | 15 | 0 | R.LVENDVAGIDVNMGCPK.Q |
| 108 – 124 | 1846.8855 | 1845.8782 | 1845.8601 | 10 | 0 | R.LVENDVAGIDVNMGCPK.Q Oxidation (M) |
| 344 – 360 | 1748.8468 | 1747.8395 | 1747.8112 | 16 | 0 | K.TSEQTGEPAEDTSGVIK.M |
| 368 – 377 | 1144.6696 | 1143.6623 | 1143.6400 | 19 | 1 | R.RAYPAQITPK.M |
| 369 – 377 | 988.5543 | 987.5470 | 987.5389 | 8 | 0 | R.AYPAQITPK.M |
| 378 - 385 | 1167.5403 | 1166.5330 | 1166.5035 | 25 | 0 | K.MCLLEWCR.R |
| 378 – 385 | 1183.5284 | 1182.5211 | 1182.4984 | 19 | 0 | K.MCLLEWCR.R Oxidation (M) |
| 389 – 399 | 1303.7378 | 1302.7305 | 1302.6932 | 29 | 0 | K.LAQPVYETVQR.P |
| 389 – 403 | 1784.9903 | 1783.9830 | 1783.9581 | 14 | 1 | K.LAQPVYETVQRPLDR.L |
| 404 – 415 | 1321.7632 | 1320.7559 | 1320.7289 | 20 | 0 | R.LFSSIVTVAEQK.Y |
| 416 – 423 | 1040.5167 | 1039.5094 | 1039.4975 | 11 | 0 | K.YQSTLWDK.S |
| 426 – 438 | 1442.8504 | 1441.8431 | 1441.8075 | 25 | 1 | K.KLAEQAAAIVCLR.S |
| 427 – 438 | 1314.7579 | 1313.7506 | 1313.7125 | 29 | 0 | K.LAEQAAAIVCLR.S |
| 439 – 446 | 843.4527 | 842.4455 | 842.4246 | 25 | 0 | R.SQGLPEGR.L |
| 447 – 456 | 1096.5810 | 1095.5738 | 1095.5560 | 16 | 0 | R.LGEESPSLHK.R |

Figure S3. Absorption spectrum of (A) 5 µM HsDus2, (B) 5 µM Dus2p, (C) 5 µM HsDus2^dusD^ and (D) 17 µM HsDus2^dsRBD^. In red is shown the purification at low ionic strength (100 mM NaCl) and in blue at high ionic strength (2 M NaCl). In the case of HsDus2, Dus2p and HsDus2^dsRBD^, purification at low ionic strength leads to an increase in absorption at 260 nm due to copurification with RNA.


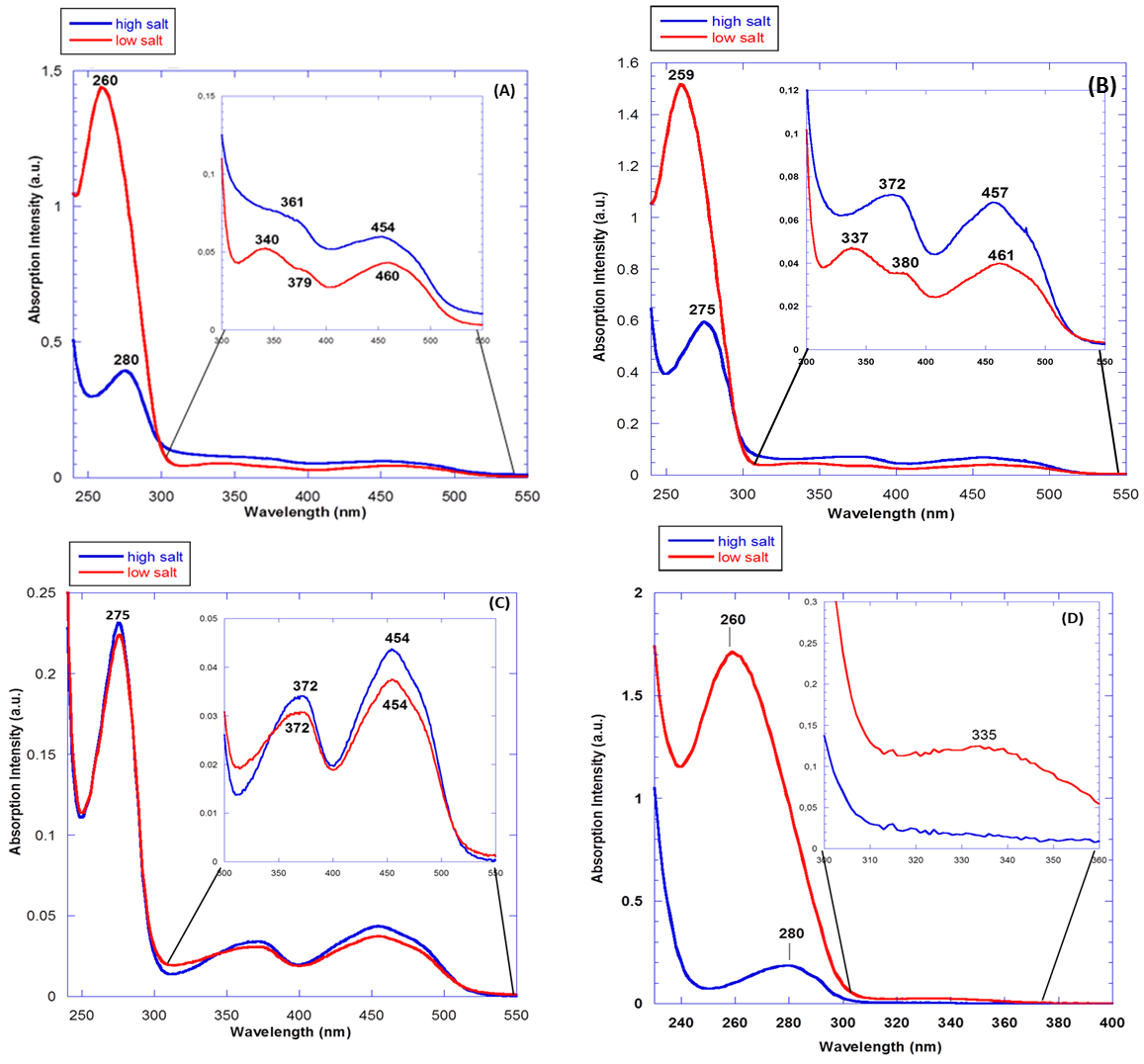


Figure S4. Size exclusion chromatography profiles of (A1) 50 µM HsDus2 purified with high salt, (A2) 50 µM HsDus2 purified with low salt, (B1) 25 µM Dus2p purified with high salt, (B2) 25 µM Dus2p purified with low salt, (C1) 25 µM HsDus2^dsRBD^ purified with high salt, (C2) 25 µM HsDus2^dsRBD^ purified with low salt, (D1) 50 µM HsDus2^dusD^ purified with high salt and (D2) 50 µM HsDus2^dusD^ purified with low salt. Absorbance at 260 nm is shown in red, at 280 nm in blue and at 450 nm in purple. Absorbance at 450 nm was multiplied by 5 in A2 and B2 for clarity. (E) Calibration curve of the SEC chromatography using gel filtration standards (globular proteins) from BIO-RAD. The table below the curve shows the calculated molecular weights of the proteins and the protein-RNA complexes based on this calibration curve. The predicted molecular weight for each protein was obtained from ExPASy-ProtParam tool. High and low salt correspond to purification of the proteins with a NaCl concentration of 0.1 and 2 M, respectively.


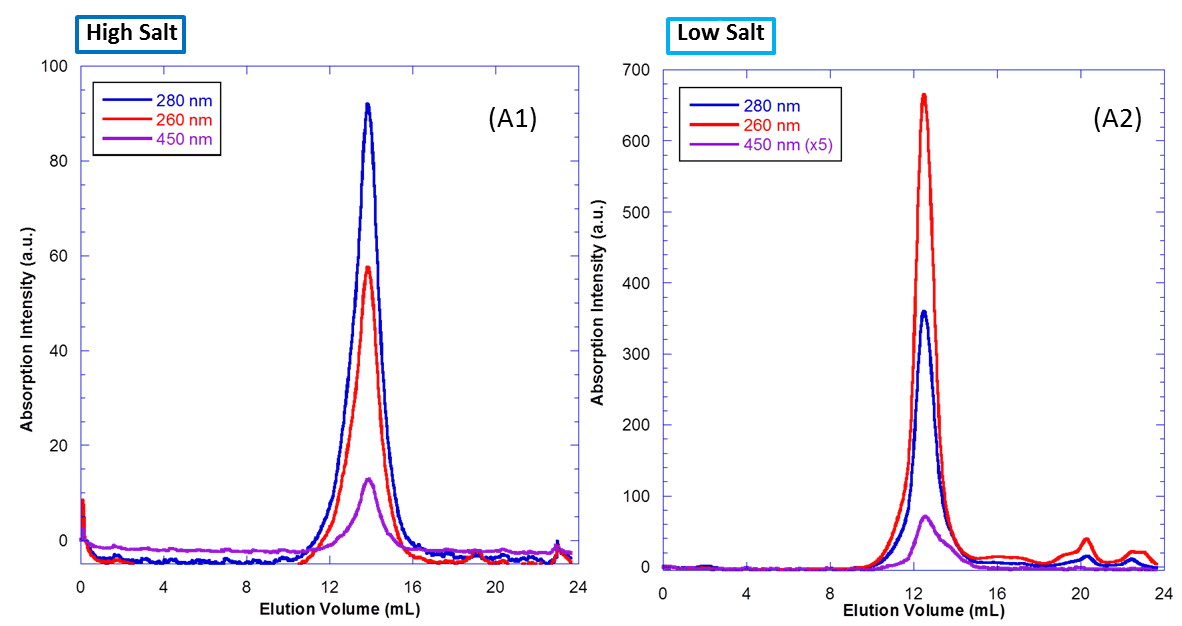


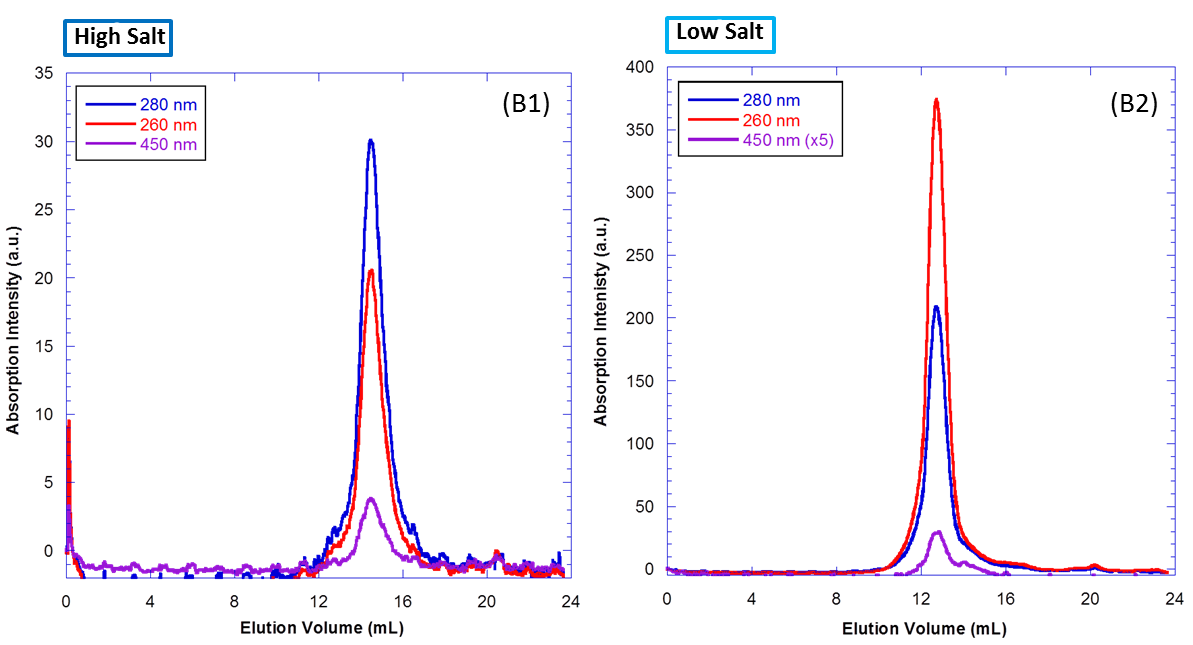


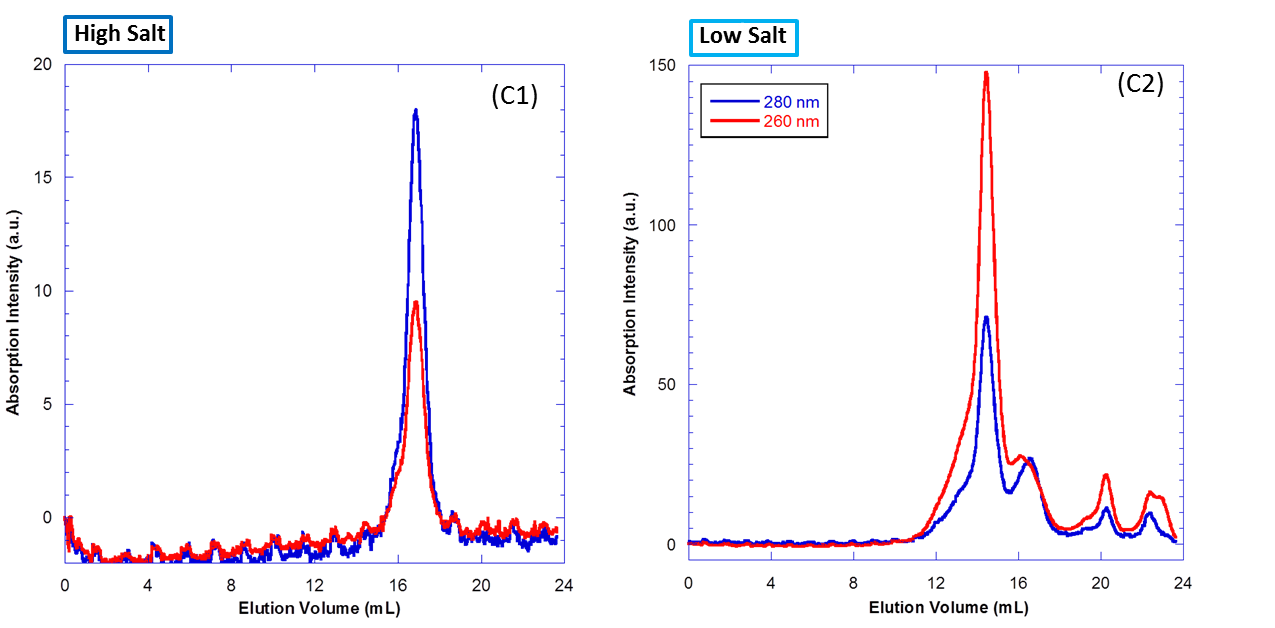


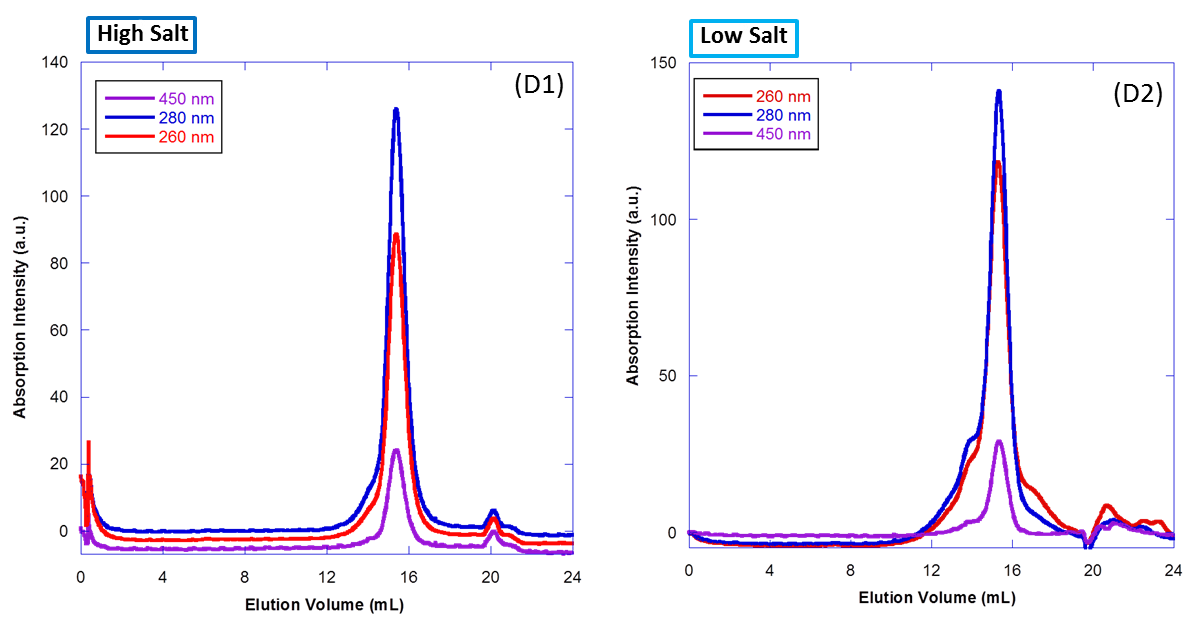


(E)


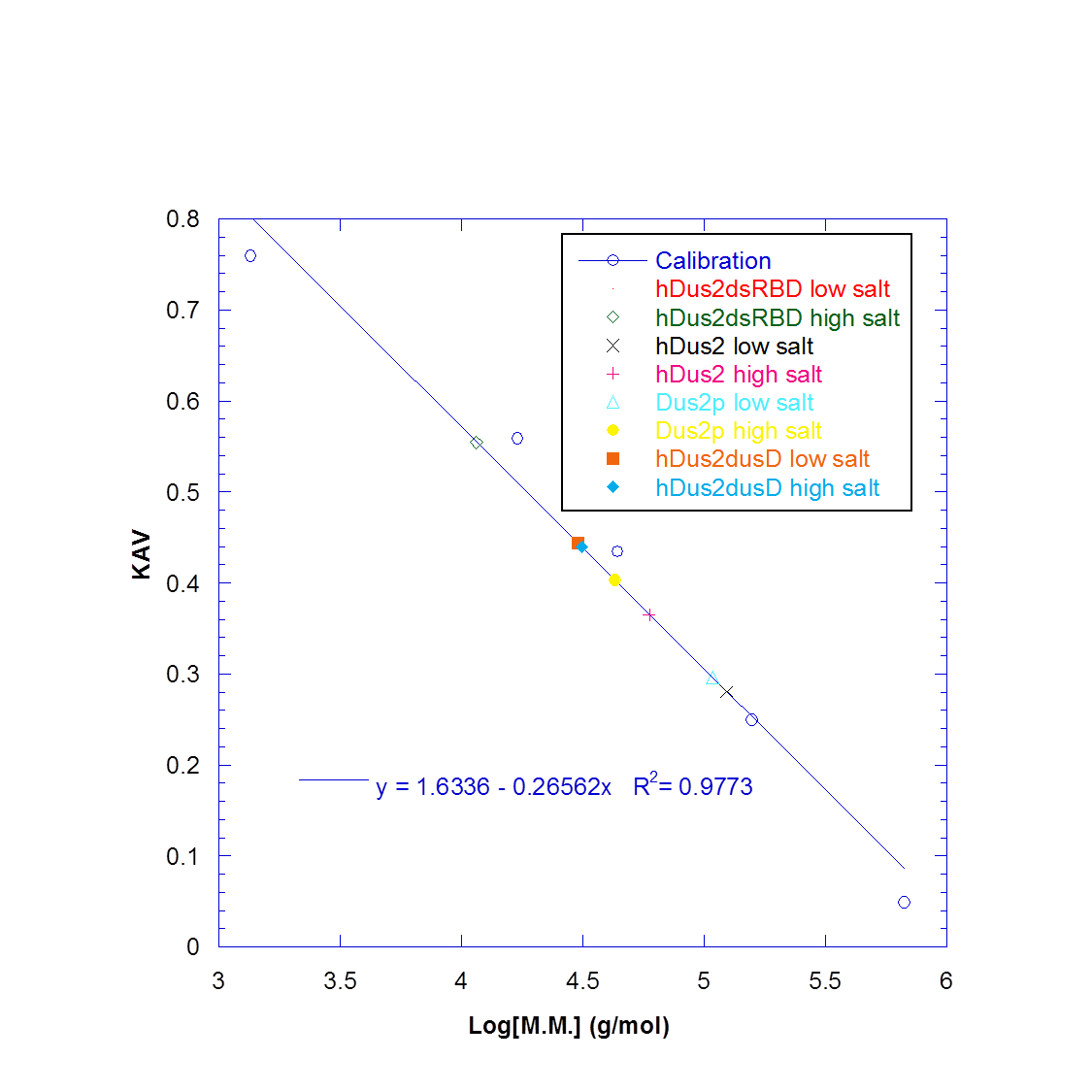

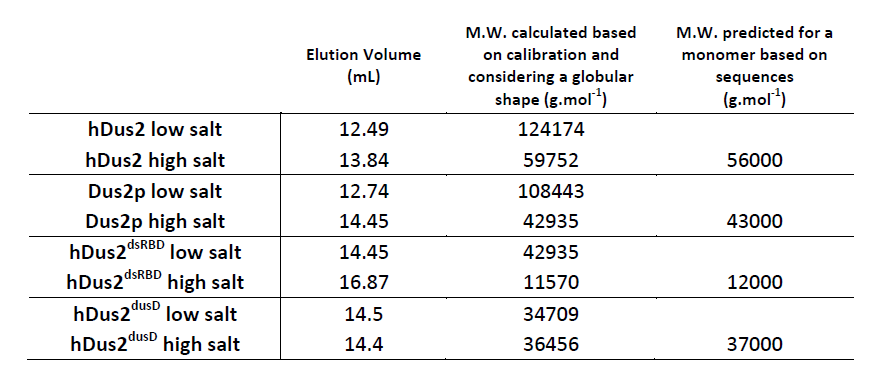


Figure S5. MALDI-MS analysis of digested tRNAs after *in vitro* assay. MALDI-MS spectrum of RNAs fragments resulting from RNAse A digestion of bulk tRNAs originated from (A) incubation of bulk Δ*dus2* tRNA in the presence of HsDus2 and NADPH, (B) bulk Δ*dus2* tRNA in the presence of HsDus2^dusD^ and NADPH, (C) bulk Δ*dus2* tRNA in the presence of HsDus2^dsRBD^ and NADPH, (D) bulk Δ*dus2* tRNA alone. Peaks are identified by their *m/z* value and the corresponding trinucleotide obtained after RNAse A digest of bulk tRNA. T is ribothymidine, " is 1-methyladenosine, L is 2-methylguanosine, K is 1-methylguanosine and # is 2'-O-methylguanosine.

**A.**


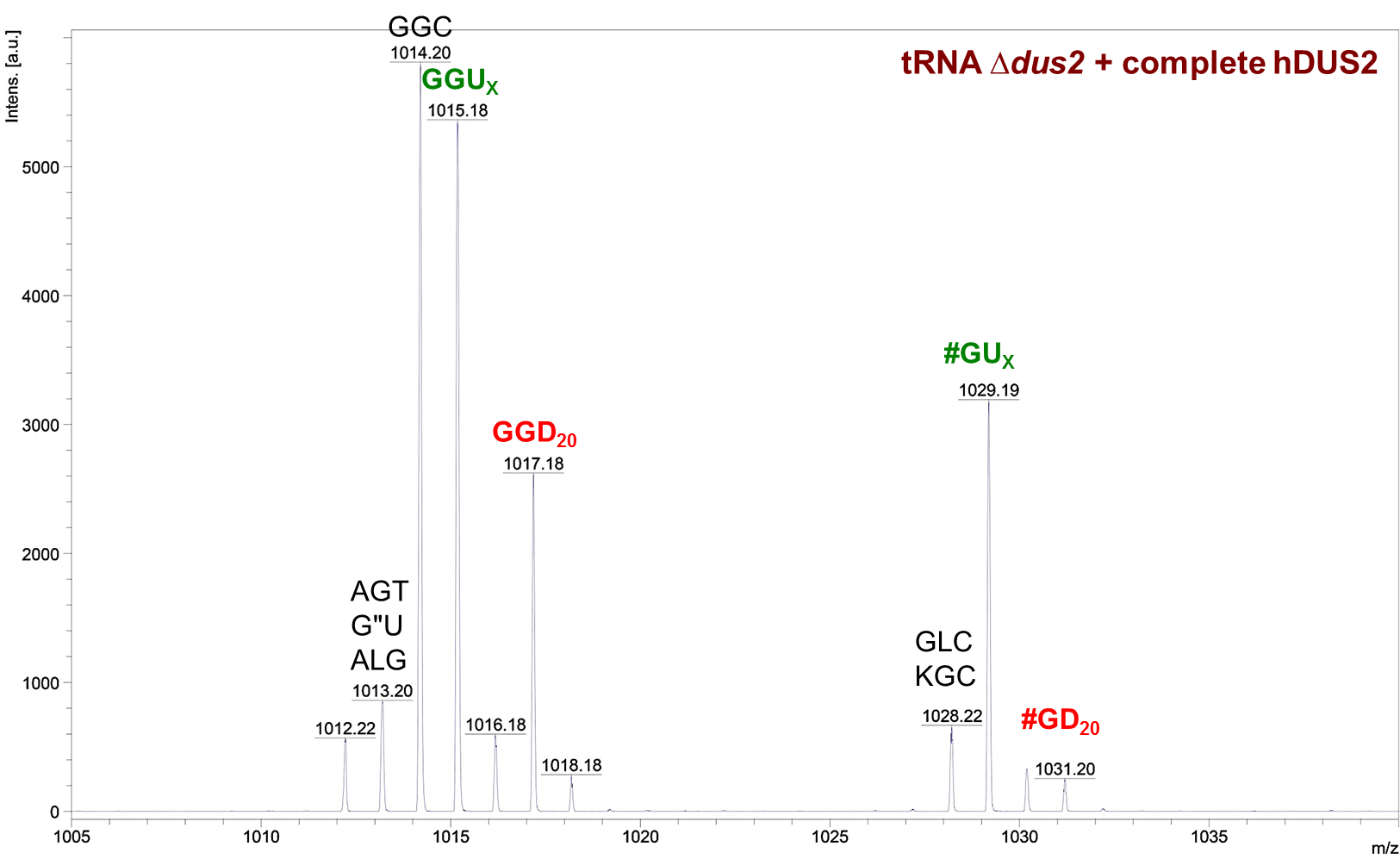


**B.**


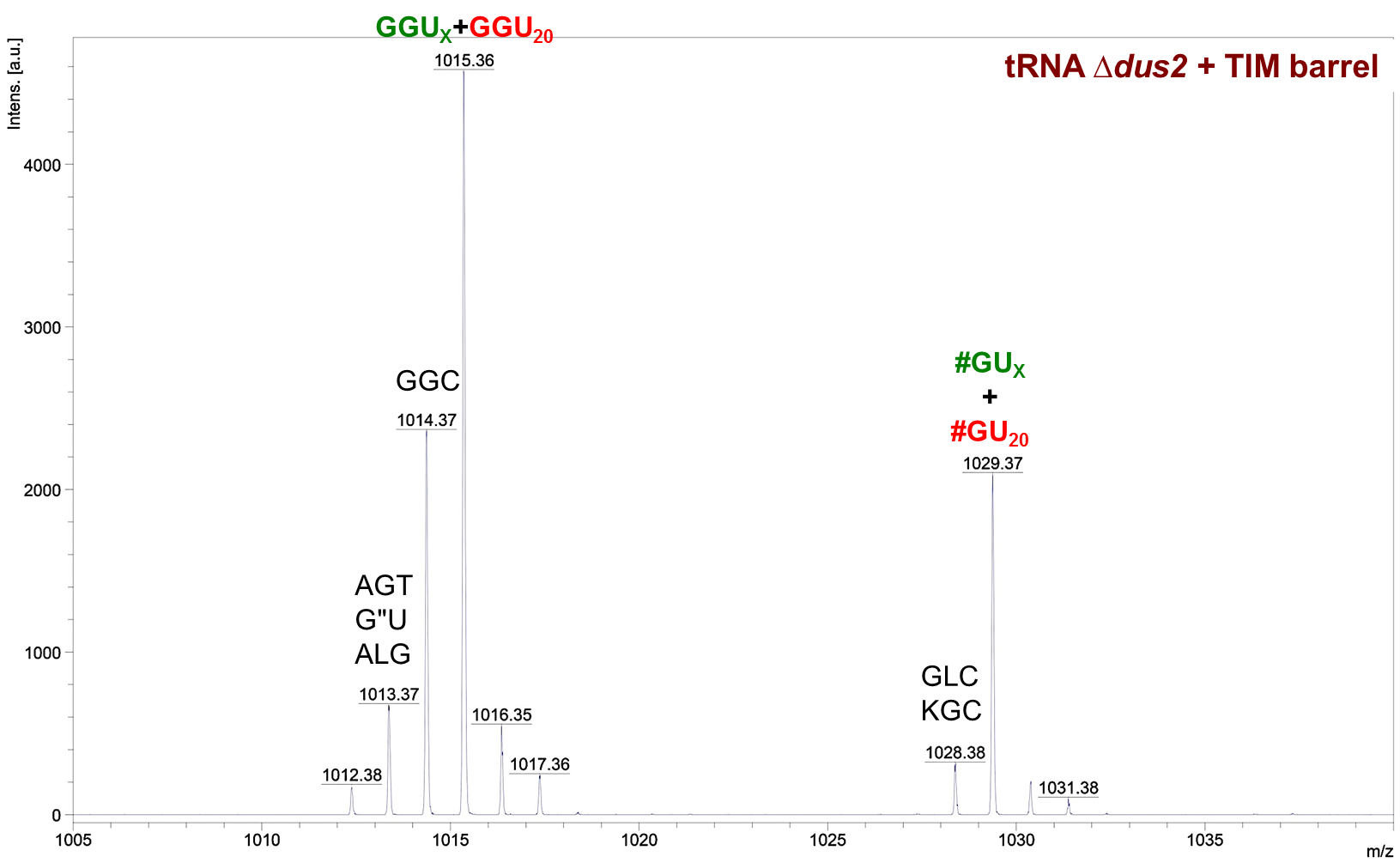


**C.**


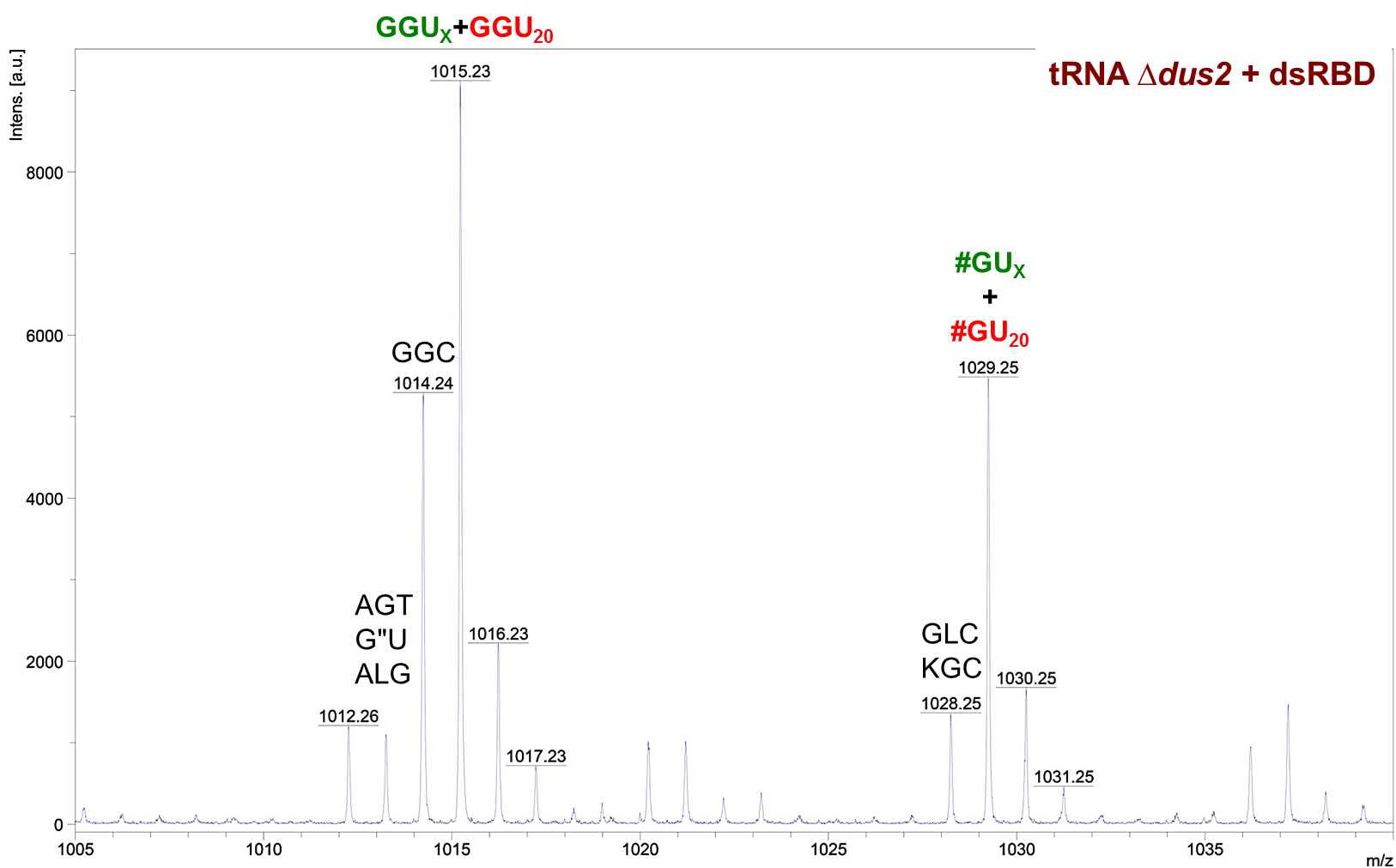


**D.**


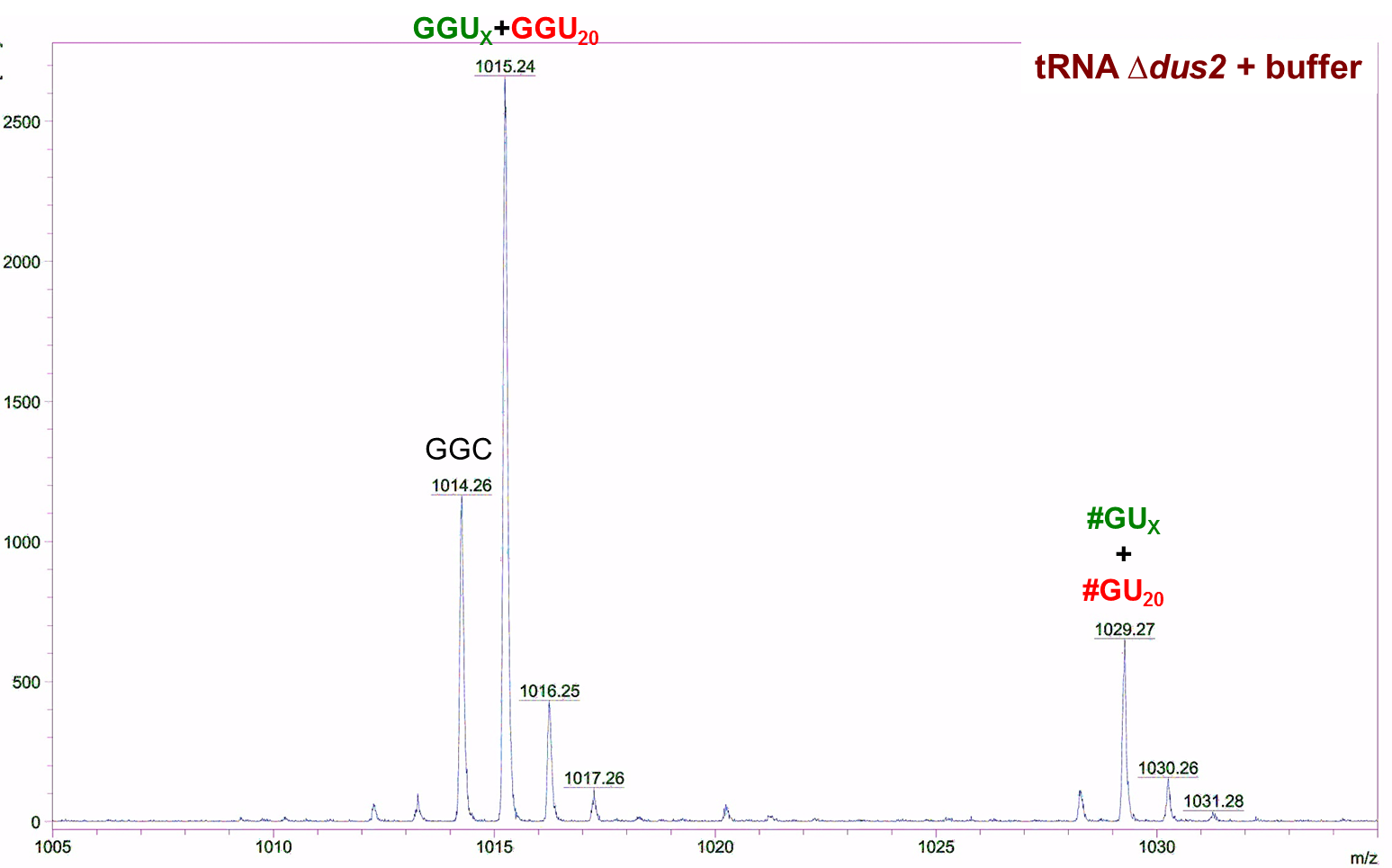


Figure S6. tRNA binding isotherm obtained from the normalized intensity of EMSA experiment. Data were fitted to a tight binding equation:

**
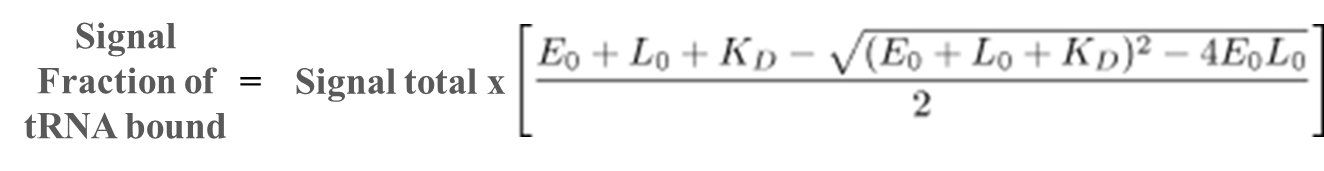
 ,** where E0 is the protein added, L0 is the concentration of tRNA and K_D_ is the dissociation constant for the protein-tRNA complex.


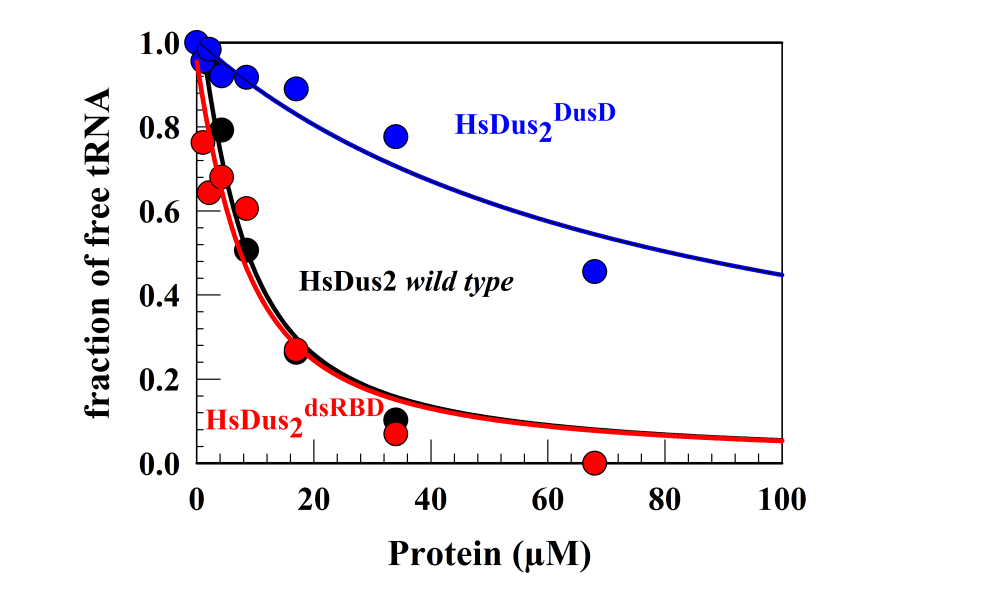


Figure S7. Ability to form stable complexes with *in vitro* transcribed yeast tRNA^Asp^ for (A) HsDus2 and (B) HsDus2^dsRBD^. Assays were carried out with 10 µM tRNA and increasing protein concentrations by a factor of 2 from 1.06 to 136 µM Protein was omitted in the first line.

**A**

**Protein**

N-

TIM BARREL

α-HELICAL

dsRBD

-C


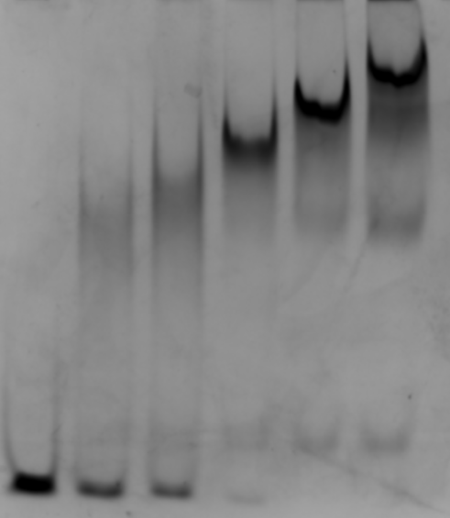


**hDUS_2_-tRNA^asp^**

**Free tRNA^asp^**


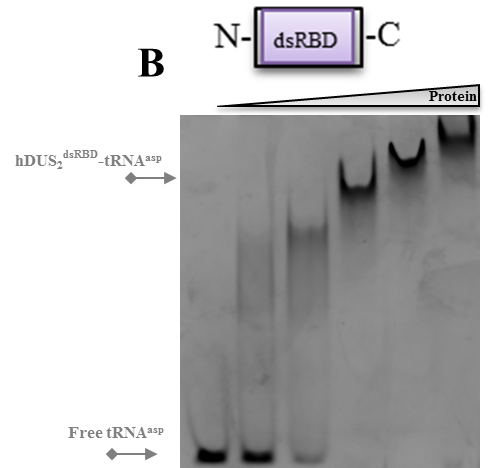


Figure S8. Representation of the topology of the individual domains of HsDus2. Secondary structure elements are labeled α for helices, β for strands and η for 3_10_ helices. The first and last residues of each secondary structure element are indicated. A) Topology of HsDus2^dusD^. The helices and strands of the TIM-barrel α8/β11 are in green and yellow, respectively. The 3 additional β strands inserted in the TIM-barrel are in red. The secondary structure elements belonging to the α-helical C-terminal domain of the TIM-barrel are in blue. B) Topology of HsDus2^dsRBD^. The color code is as in figure 4. The N-terminal extension is in magenta. The figure was prepared with Pro-origami ([18](#_ENREF_18))


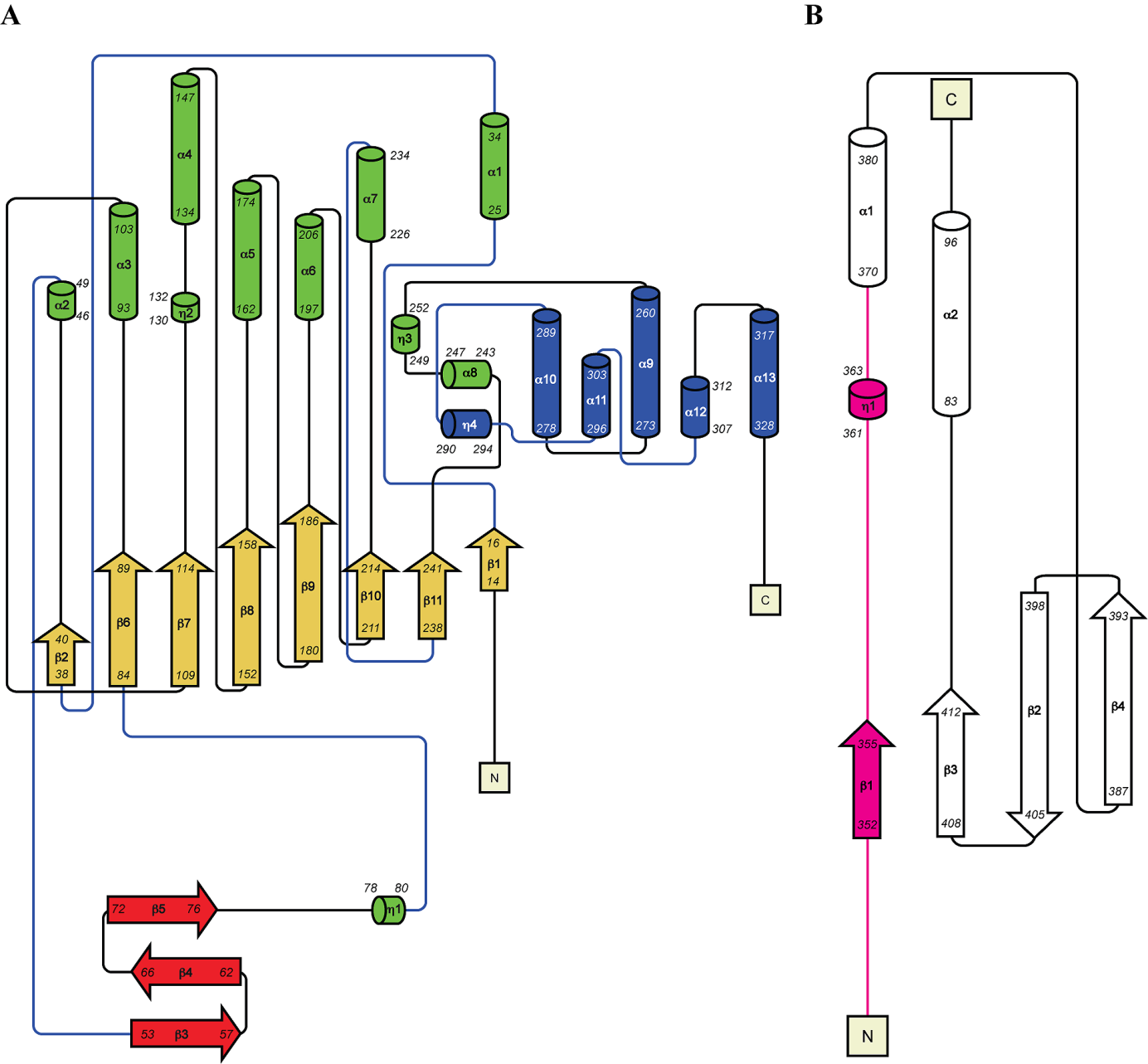


Figure S9. Overview of the FMN binding site of HsDus2. (A) A 2Fo-Fc σA-weighted electron density map of the FMN binding site, omitting FMN and contoured at 1 sigma, is shown in green. This density is best fitted with two different conformations of FMN, shown in yellow and pink sticks, which are detailed in B and C. B) Second conformation (44%) observed in our X-ray structure. This conformation is represented in pink. Potential hydrogen bonds between FMN and the TIM-barrel of HsDus2 are displayed as dashes. C) Overview of the main conformations of the FMN. The main conformation (56%) is in yellow. The isoalloxazine rings are identical in both conformations. Changes are localized on the C4’, C5’ of the ribityl chain and the phosphate group.


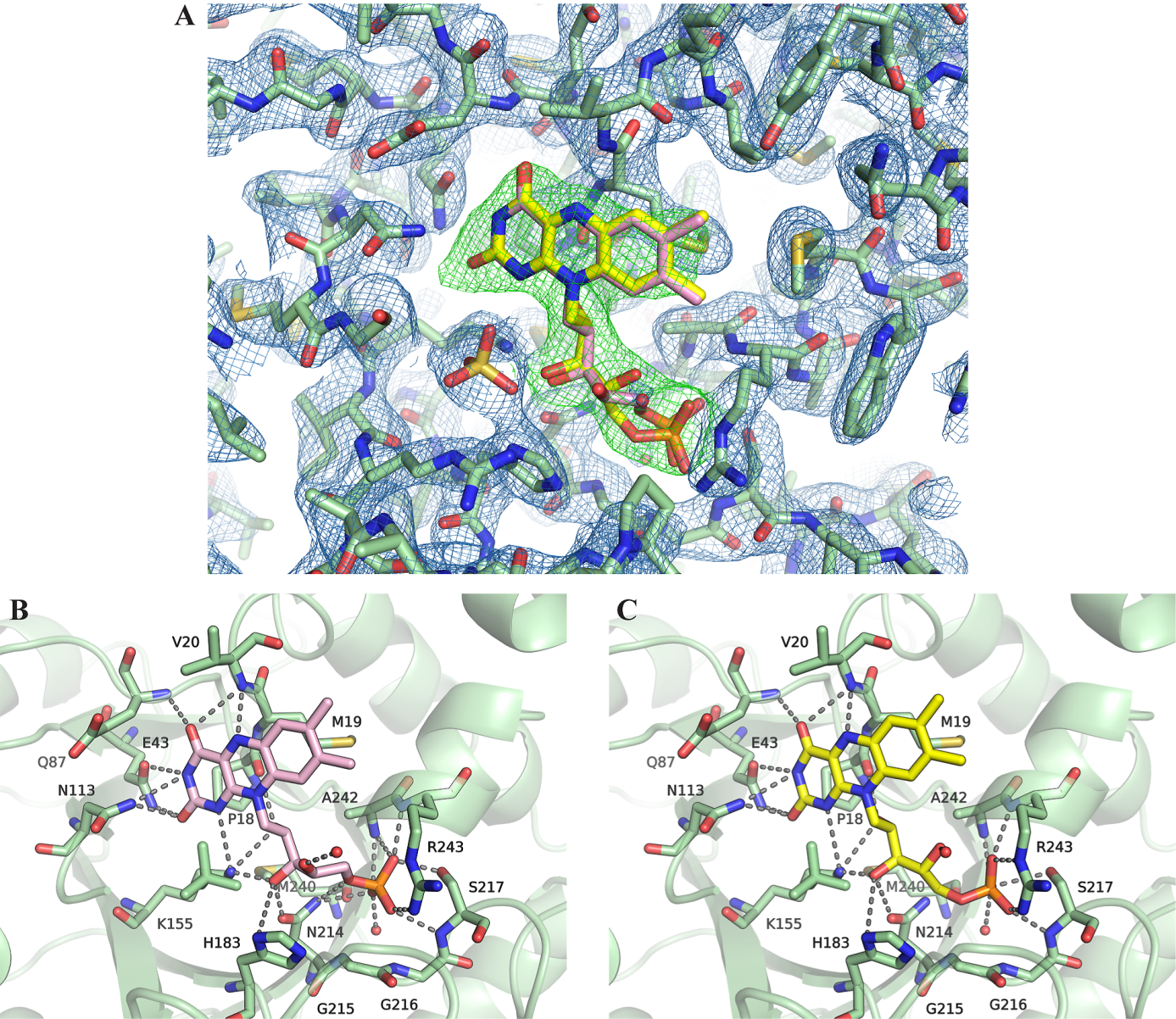


Figure S10. Comparison of the crystal structures of the TIM-barrel and the helical domains of the Dus proteins: (A) HsDus2^dusD^ (B) *T. thermophilus* (pdb 3b0p), (C) *E.* *coli* (pdb 4bfa) and (D) *T.* *maritima* (pdb 1vhn).


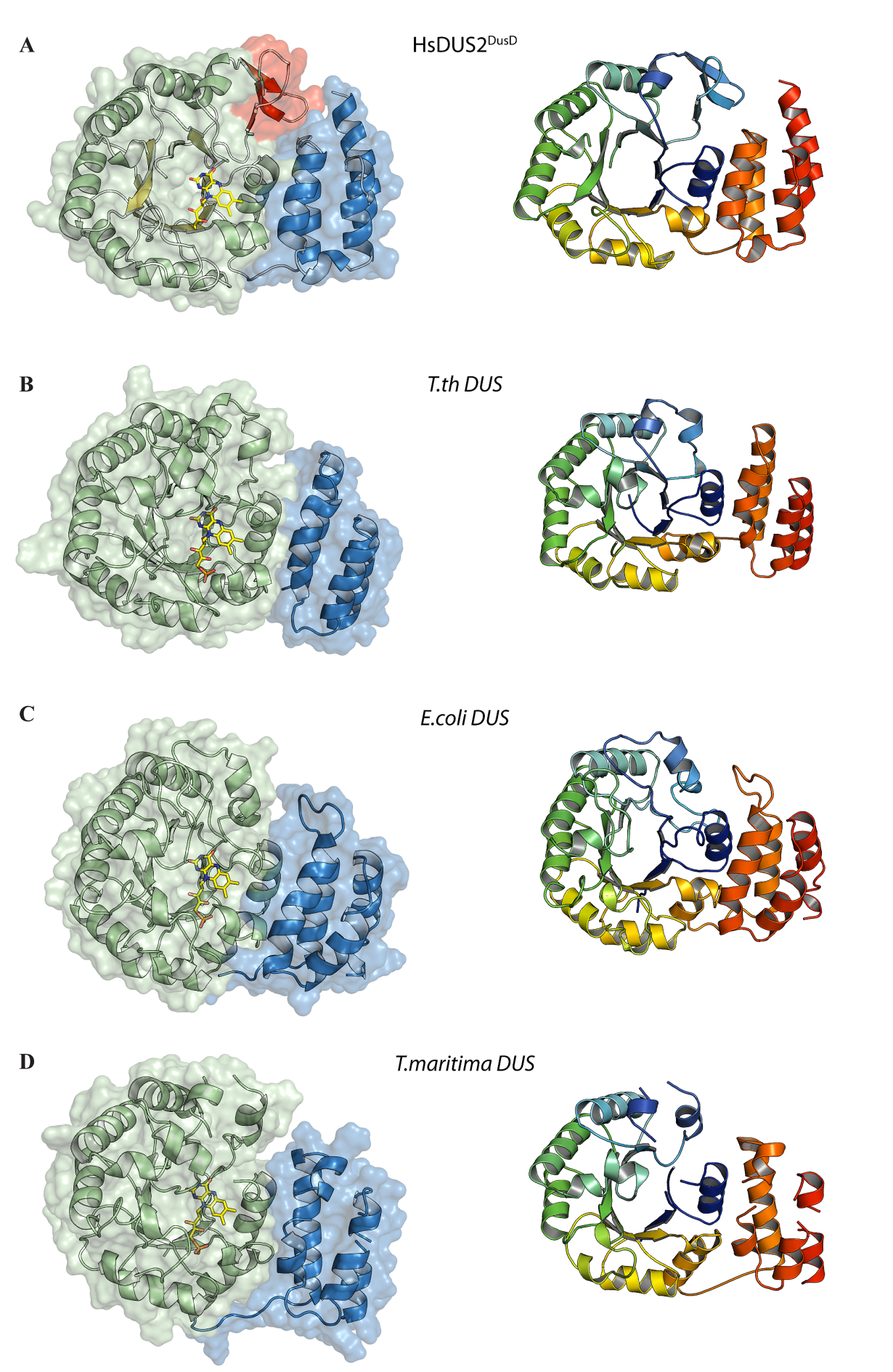


Figure S11. (A) Stereo-view of structural alignment of the TIM-barrel domain of HsDus2^dusD^ (green) *T. thermophilus* (cyan), *E.* *coli* (magenta) and *T.* *maritima* (orange). (B) Plots representing the RMSD versus residue resulting from the structural alignment of HsDus2^dusD^ with TthDus (B) or with EcDusC (C) or TmDus (D).

Figure S12. (A) Sequence alignment of HsDus2^dsRBD^ with various double-stranded RNA binding domains from fly fruit (*Dorsophila melanogaster*, Dm), human (*Homo sapiens*, Hs), frogs (*Xenopus leavis*, XI), baker’s yeast (*Saccharomyces cerevisiae*, Cs), bacteria (*Escherichia coli*, Ec and *Aquifex aeolicus*, Aa) and plants (*Arabidopsus thaliana*, At). Alignment was done by ClustalW2 and manually adjusted. The consensus sequence (>40%) is shown below the alignment. (B) Structure of different dsRBDs. In the center is represented HsDus2^dsRBD^; *X. leavis* RBPA (1DI2) and *A. aeolicus* (2NUG) dsRBDs are at the top left and right hand sides, respectively. The dsRBDs of *A. thaliana* HYL1 (3ADG) and *human* ADAR1-dsRBD_3_ (2MDR) are at the bottom left and right hand sides, respectively. The extension structure of the dsRBDs is indicated in yellow.

**(A)**


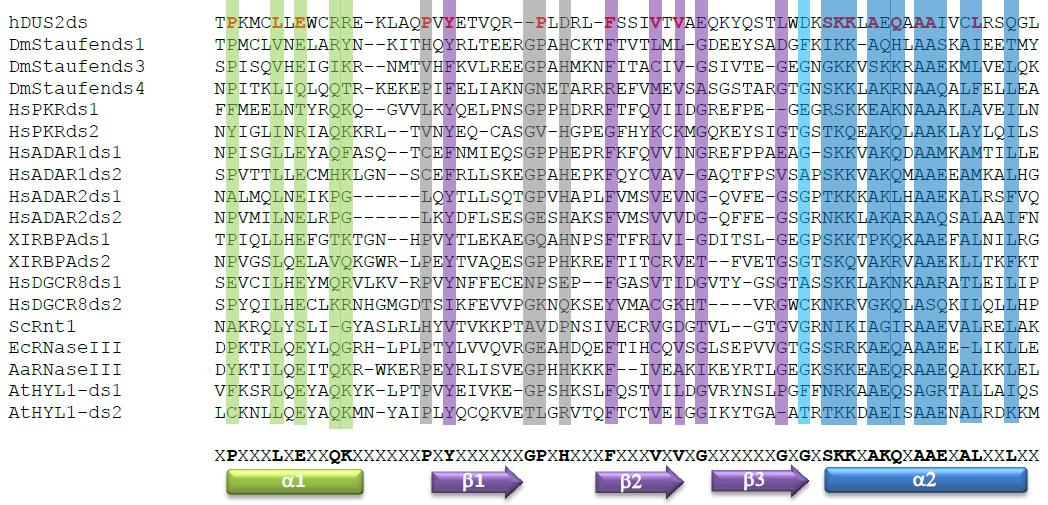


**(B)**


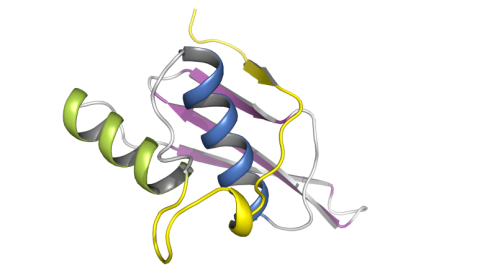

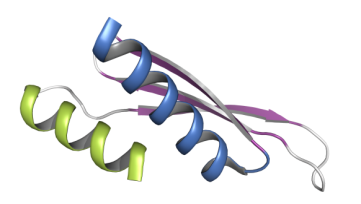

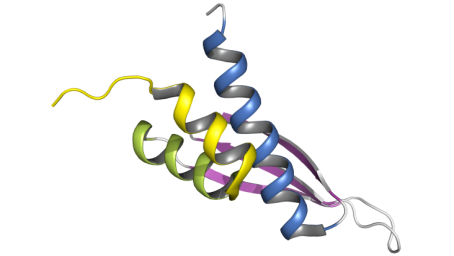

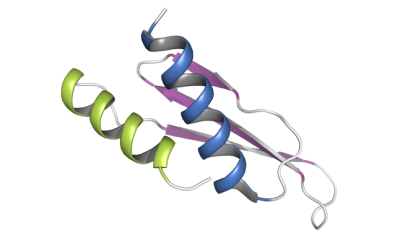

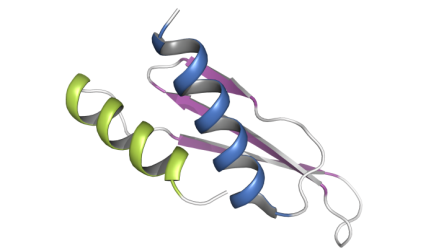


Table S1. Summary of data collection and refinement statistics

| **Data collection** | dsRBD S-SAD | dsRBD Native | TIM-BARREL |
| --- | --- | --- | --- |
| Space group | P4_3_ | P4_3_ | I222 |
| Unit-cell parameters (Å) | *a* = 55.49; *b* = 55.49;  *c* = 115.22 | *a* = 55.13; *b* = 55.13;  *c* = 114.66 | *a* = 71.57; *b* = 85.26;  *c* = 144.23 |
| Wavelength (Å) | 1.771 | 1.000 | 0.9801 |
| Temperature (K) | 100 | 100 | 100 |
| No. of crystals | 1 | 1 | 1 |
| Resolution range (Å)  (Highest resolution shell range) | 49.99 – 2.20  (2.279 – 2.20) | 39.74 – 1.70  (1.75 – 1.69) | 43.64 – 2.68  (2.77 – 2. 68) |
| Total no. of reflections | 743592 (65450) | 172181 (15833) | 91491 (8905) |
| No. of unique reflections | 17688 (1748) | 38156 (3699) | 12686 (1250) |
| Completeness (%) | 100 (100) | 99.69 (97.65) | 99.09 (91.76) |
| I/σ(I) | 86.30 (18.76) | 22.06 (4.16) | 19.87 (2.69) |
| *R*_meas_ | 0.044 | 0.049 | 0.092 |
| **Refinement** |  |  |  |
| *R*_work_/*R*_free_[*^a^*](http://www.jbc.org.gate1.inist.fr/content/288/6/4095/T1.expansion.html#fn-2) | 0.16 / 0.23 | 0.16 / 0.18 | 0.19/0.24 |
| Molecules per asymmetric unit | 3 | 3 | 1 |
| No. of amino acid residues | 277 | 281 | 306 |
| No. of ligands | - | - | 3 |
| No. of water molecules | 200 | 367 | 28 |
| r.m.s. deviations |  |  |  |
| Bond lengths (Å) | 0.018 | 0.007 | 0.011 |
| Bond angles (°) | 1.93 | 1.005 | 1.28 |
| Ramachandran plot (%) |  |  |  |
| Most favored | 99 | 100 | 97 |
| Disallowed | 0.74 | 0 | 0 |
| Molprobity clashscore |  |  | 5.25 |
| Average *B* factors (Å^2^) |  |  |  |
| protein | 30.20 | 29.70 | 54.10 |
| Water | 33.00 | 39.20 | 57.50 |
| Ligands | - | - | 47.70 |
| Overall | 30.40 | 31.00 | 53.90 |
| Wilson B factor | 26.59 | 21.38 | 54.90 |
| Coordinate error (Å) | 0.13 | 0.15 | 0.34 |
|  |  |  |  |
|  |  |  |  |
|  |  | |  |

The highest resolution shells with 5% of the data are shown in parentheses. R_meas_ defined as:

Table S2. Kinetics parameters for NADP(H) oxidation by HsDus2

**Kinetics with NADH Kinetics with NADPH**

k_cat_  K_M_ k_cat_/K_M_  k_cat_  K_M_ k_cat_/K_M_

*s^-1^ μM 10^4^ M^-1^.s^-1^ s^-1^ μM 10^4^ M^-1^.s^-1^*

hDUS2 1 ± 0.15 123 ± 13 0.8 ± 0.01 1.9 ± 0.2 41 ± 3 4.6± 0.07

TIM Barrel 0.9 ± 0.1 126 ± 15 0.7 ± 0.007 1.3 ± 0.15 41 ± 5 3.2 ± 0.03

TIM Barrel + dsRBD 0.7 ± 0.05 137 ± 17 0.5 ± 0.003 1.2 ± 0.1 42 ± 3 2.8 ± 0.03

REFERENCES

1. Kasprzak JM, Czerwoniec A, & Bujnicki JM (2012) Molecular evolution of dihydrouridine synthases. *BMC bioinformatics* 13:153.

2. Kao C, Zheng M, & Rudisser S (1999) A simple and efficient method to reduce nontemplated nucleotide addition at the 3 terminus of RNAs transcribed by T7 RNA polymerase. *RNA* 5(9):1268-1272.

3. Hamdane D*, et al.* (2011) Insights into folate/FAD-dependent tRNA methyltransferase mechanism: role of two highly conserved cysteines in catalysis. *The Journal of biological chemistry* 286(42):36268-36280.

4. Kabsch W (2010) Xds. *Acta crystallographica. Section D, Biological crystallography* 66(Pt 2):125-132.

5. Terwilliger TC*, et al.* (2012) phenix.mr_rosetta: molecular replacement and model rebuilding with Phenix and Rosetta. *Journal of structural and functional genomics* 13(2):81-90.

6. DiMaio F, Tyka MD, Baker ML, Chiu W, & Baker D (2009) Refinement of protein structures into low-resolution density maps using rosetta. *Journal of molecular biology* 392(1):181-190.

7. Soding J (2005) Protein homology detection by HMM-HMM comparison. *Bioinformatics* 21(7):951-960.

8. Cowtan K (2006) The Buccaneer software for automated model building. 1. Tracing protein chains. *Acta crystallographica. Section D, Biological crystallography* 62(Pt 9):1002-1011.

9. Emsley P, Lohkamp B, Scott WG, & Cowtan K (2010) Features and development of Coot. *Acta crystallographica. Section D, Biological crystallography* 66(Pt 4):486-501.

10. Bricogne G*, et al.* (2011) BUSTER version 2.10.0. Cambridge, United Kingdom: Global Phasing Ltd.

11. Sheldrick GM (2008) A short history of SHELX. *Acta crystallographica. Section A, Foundations of crystallography* 64(Pt 1):112-122.

12. Adams PD*, et al.* (2010) PHENIX: a comprehensive Python-based system for macromolecular structure solution. *Acta Crystallogr D Biol Crystallogr* 66(Pt 2):213-221.

13. Cowtan K (2010) Recent developments in classical density modification. *Acta crystallographica. Section D, Biological crystallography* 66(Pt 4):470-478.

14. Murshudov GN*, et al.* (2011) REFMAC5 for the refinement of macromolecular crystal structures. *Acta crystallographica. Section D, Biological crystallography* 67(Pt 4):355-367.

15. Adams PD*, et al.* (2010) PHENIX: a comprehensive Python-based system for macromolecular structure solution. *Acta crystallographica. Section D, Biological crystallography* 66(Pt 2):213-221.

16. Rider LW, Ottosen MB, Gattis SG, & Palfey BA (2009) Mechanism of dihydrouridine synthase 2 from yeast and the importance of modifications for efficient tRNA reduction. *The Journal of biological chemistry* 284(16):10324-10333.

17. Yu F*, et al.* (2011) Molecular basis of dihydrouridine formation on tRNA. *Proceedings of the National Academy of Sciences of the United States of America* 108(49):19593-19598.

18. Stivala A, Wybrow M, Wirth A, Whisstock JC, & Stuckey PJ (2011) Automatic generation of protein structure cartoons with Pro-origami. *Bioinformatics* 27(23):3315-3316.

19. Baker NA, Sept D, Joseph S, Holst MJ, & McCammon JA (2001) Electrostatics of nanosystems: application to microtubules and the ribosome. *Proceedings of the National Academy of Sciences of the United States of America* 98(18):10037-10041.

20. Sundaralingam M, Rao ST, & Abola J (1971) Molecular conformation of dihydrouridine: puckered base nucleoside of transfer RNA. *Science* 172(3984):725-727.
